# Microbial conversion of vegetable waste for flavor additives via solid-state fermentation: a comprehensive review

**DOI:** 10.3389/fnut.2025.1445189

**Published:** 2025-06-24

**Authors:** Janani Latha Ravi, Payel Ghosh, Faraz Ahmad, Shafiul Haque, Paula Barciela, Franklin Chamorro, Ana Olivia Serra Jorge, Miguel A. Prieto, Sandeep Singh Rana

**Affiliations:** ^1^Department of Bioscience, School of BioSciences and Technology, Vellore Institute of Technology, Vellore, India; ^2^Department of Food Technology, School of Agriculture and Food Technology, Vignan’s Foundation for Science, Technology and Research, Guntur, India; ^3^Department of Biotechnology, School of Bio Sciences and Technology, Vellore Institute of Technology, Vellore, India; ^4^Research and Scientific Studies Unit, College of Nursing and Health Sciences, Jazan University, Jazan, Saudi Arabia; ^5^School of Medicine, Universidad Espiritu Santo, Samborondon, Ecuador; ^6^Universidade de Vigo, Nutrition and Food Group (NuFoG), Department of Analytical Chemistry and Food Science, Instituto de Agroecoloxía e Alimentación (IAA) – CITEXVI, Vigo, Spain; ^7^REQUIMTE/LAQV, Department of Chemical Sciences, Faculty of Pharmacy, University of Porto, Porto, Portugal

**Keywords:** agro-industrial waste, flavor, additives, solid-state fermentation, microbial biotransformation

## Abstract

Flavor is a key factor in consumer choice and food acceptance. Currently, the vast majority of food flavor additives are produced by chemical synthesis. However, alternative production methods have been explored to meet consumer demands for “clean label” foods and “natural” additives. Consumer demand for natural products and the need for environmentally friendly processes are driving the development of novel biotechnology-based technologies for flavor synthesis. The bioproduction of dietary flavor molecules using plant waste has emerged as a viable possibility. This *de novo* synthesis in flavor production offers a way to create unique and desirable flavor compounds that are not readily available from natural sources. This study focuses on the creation of flavor molecules through microbial biotransformation, with particular emphasis on Solid-State Fermentation (SSF). SSF is a fermentation method in which microorganisms grow on a solid material without free-flowing water. Several microorganisms are used in SSF to produce flavor compounds, including the most commonly used fungi, but also lactic acid bacteria and yeast. The use of abundant and inexpensive vegetable waste produced by agro-industrial processing systems as a viable substrate for microbial flavor chemical production by SSF is highly encouraged from both sustainability and cost efficiency perspectives. Therefore, this review can serve as a basis for further studies aimed at developing effective and low-cost technologies for the extraction of essential flavors from agricultural residues.

## 1 Introduction

Flavor is a fundamental aspect of food perception, directly influencing consumer acceptance and preferences. It arises from the combination of olfactory and gustatory sensations, with taste being determined by non-volatile molecules interacting with tongue receptors, while aroma is perceived through volatile organic compounds (VOCs) detected in the nasal cavity (1, 173). Among these VOCs, terpenes, esters, and aldehydes play a key role in defining food aroma, which significantly impacts the sensory appeal of food products and drives innovation in the food industry (2). Traditionally, the majority of flavor compounds have been synthesized chemically, often from petroleum-derived feedstocks. However, growing consumer demand for “clean label” products, combined with increasing environmental concerns, has led to a shift toward sustainable and natural flavor production methods (3, 4). One promising alternative is biotechnological flavor production, where microbial fermentation is used to convert inexpensive substrates into valuable aroma compounds. Agricultural by-products, which are nutrient-rich but often discarded as waste, present an economically viable and sustainable raw material for microbial fermentation (5).

Among biotechnological approaches, solid-state fermentation (SSF) has gained significant attention as an efficient method for extracting natural flavors from agri-food waste (11). Unlike traditional sumerged fermentation (SmF), SSF enables microbial growth on solid substrates without free-flowing water, closely mimicking natural fermentation environments. This process has been successfully applied to transform agricultural residues such as sugarcane bagasse (SCB), coconut husks, and other vegetable by-products into high-value flavor and fragrance compounds (12, 13).

This review explores the potential of microbial biotransformation for flavor production, with a specific focus on SSF as a sustainable approach for utilizing vegetable waste. By examining the role of different microorganisms, fermentation conditions, and substrate selection, this review aims to highlight the feasibility of SSF in generating value-added food additives. Additionally, the challenges and future directions in this field will be discussed to support the development of eco-friendly, cost-effective flavor production technologies.

## 2 Flavor additives

Food additives include colorants, preservatives, antioxidants, sweeteners, emulsifiers, stabilizers, thickeners, and gelling agents, used to preserve flavor, enhance food taste, appearance, or other qualities (14). In contrast, flavor additives include compounds such as organic acids, humectants, mineral salts, and low-caloric and high-intensity sweeteners (9). These can be artificial, such as most vanillin compounds and the banana-flavored isoamyl acetate, or natural, such as flavor extracts, spices and herbs. It should be noted that some additives are not used exclusively for flavor, as they often have multiple functions (15). For example, sorbitol, a sugar alcohol, is both a humectant and a sweetener. Citric acid is also both a preservative, lowering the pH of the food, and a flavoring agent (16).

Flavor can significantly affect customer pleasure and subsequent food intake (17). Consumers often associate flavor with other product features, including appearance, acidity, salt, and sweetness (18). Research indicates flavor-enhanced food can improve palatability, boost salivary flow and immunity, and minimize chemosensory complaints in older people (19). However, taste improvement is not the only use of food flavoring additives. One of the most important uses is to mask off-flavors, covering up undesirable tastes that might arise from certain ingredients or processing methods, ensuring a more pleasant eating experience (20). Flavor additives are also employed to help maintain a consistent flavor profile across different batches of food, ensuring that consumers receive the same taste experience every time. These additives can also replace undesirable ingredients such as sugar or salt, producing low-calorie or low-sodium foods (7). This is the case for monosodium glutamate, which can enhance the savory taste of food and allow for reduced sodium content without compromising flavor. Additionally, with the demand for specific taste profiles varying by region and culture, flavor enhancers can be used to tailor products to specific consumer preferences (21). A listing of flavor compounds, their classification, sensory characteristics and applications can be found on Table 1.

**Table 1:** Classification of flavor compounds: examples, sensory characteristics, and applications.

Category	Examples	Sensory features	Application	Microorganism	Ref.
Aldehydes	2-methylbutanal	Sweet, caramel, and nutty	Flavor enhancers and fragrance compounds	*Sphingobium* sp.	Li et al. (163)
Lactone	Γ-dodecalactone and δ-decalactone	Fruity, milky, coconut, and other aromas	Flavoring agents and dairy products	*Saccharomyces cerevisiae*	Marella et al. (164)
Alcohol	2-phenylethanol	Rose	Perfumes and beverages	*Pichia kudriavzevii*	Martínez-Avila et al. (165)
Fatty acids	Propionic acid	Sour	Cheese and sourdough bread	*Kluyveromyces marxianus*	Smit et al. (166)
Terpenes	Α-terpineol	Floral and lilac-like aroma	Perfume and air fresheners	*Trichoderma harzianum*	Molina et al. (167)
Pyrazines	2,5-dimethyl pyrazine	Roasted and nutty taste	Roasted nuts and coffee	*Enterobacter hormaechei*	Yang et al. (168)
Esters	Ethyl Acetate	Sweet	Artificial fruit flavors and solvent	*Kluyveromyces marxianus*	Correia-Lima et al. (138)
Ketones	Diacetyl	Creamy taste	Butter flavor, dairy products	*Saccharomyces cerevisiae*	Desnoyers et al. (71)

### 2.1 Natural flavor additives

Natural food additives are substances derived from natural sources such as plants, animals, and minerals (175). These offer a more authentic and often healthier alternative to artificial additives. For example, essential oils are concentrated extracts from plants that capture their flavor and aroma. In the food industry, they are used in beverages, baked goods, and confectionery to impart a robust and natural taste (176). Essential oils regarded as safe for consumption include some citrus oils (lemon, orange), peppermint oil, and clove oil. It is to note that not all essential oils are safe for consumption as a more regulated extraction is necessary to create food-grade essential oils (22).

The most widely used natural additives are plant extracts like vanilla, almond, and mint extract. These extracts are often obtained by soaking raw materials in solvents like alcohol or water over long periods of time and at a certain temperature (23). Vanilla extract, for example, is a staple in baking and desserts for its rich, sweet flavor (24). Spices and herbs can also be classified as natural additives. Since ancient times, they have been used as food flavor additives. Examples include cinnamon, nutmeg, oregano, basil, among many others (25). They are used to enhance the flavor of a wide range of dishes. Industrially, they are often added to savory and sweet foods to provide depth and complexity in flavor (26).

Natural flavors are derived from various plant and animal sources using different extraction techniques (27). The three primary techniques are steam distillation, solvent extraction, and cold pressing. The process of steam distillation involves the use of steam to vaporize the VOCs present in the raw material. Then, the vapors are collected and condensed. Steam distillation is a widely used method for extracting essential oils from peppermint and ginger. This technique efficiently isolates volatile flavor compounds without causing thermal degradation (28). In hydrodistillation, plant material is boiled with water, causing VOcs to be carried away with the steam and condense. The VOCs are then separated from the water and dried over anhydrous sodium sulfate (29). Hydrodistillation is not commonly practiced in the industry because of the long distillation time and the resulting mass not being readily amenable for oleoresin extraction with solvents (28). In the solvent extraction method, solvents like ethanol or hexane are used to dissolve the flavor compounds from the raw material. The solvent is then evaporated, leaving behind the concentrated flavor extract. Solvent extraction is effective for VOC and non-volatile compounds non-VOCs avoid contamination (30). Cold pressing is a mechanical process primarily used for extracting citrus oils. The peel is punctured and pressed to release the oils, which are then separated from the juice and other components. Cold pressing preserves the integrity and quality of the oils, making it suitable for sensitive compounds (31).

Finally, fermented products are widely used as additives in the industry, although they are more common in Asia. These include soy sauce, miso, vinegar and nutritional yeast powder. These fermented products are rich in umami and complex flavors and can be used to enhance the savoriness of flavors or to increase the tanginess, as in the case of vinegar (32). Nutritional yeast is also often used to give a “cheesy” flavor to plant-based cheese or as a lower cost cheesy flavor in snacks (33).

### 2.2 Artificial flavor additives

Artificial flavor additives are chemically synthesized compounds that mimic or enhance the taste and aroma of natural flavors (34). Additives can be categorized into synthetic aromas and flavor enhancers. Synthetic aromas are chemically derived compounds that replicate the smell and taste of natural flavors. They are created through chemical synthesis rather than being extracted from natural sources (35). Examples of synthetic aromas include the vanilla-flavored vanillin, the banana-flavored isoamyl acetate, the grape-flavored methyl anthranilate and the cherry flavored benzaldehyde (36). The synthesis of vanillin, which is chemically identical to the major flavor component of vanilla beans, can be made chemically from guaiacol and glyoxylic acid or by oxidation of lignin. The process involves several steps, including acetylation of isoeugenol to isoeugenol acetate, oxidation to vanillin acetate, and hydrolysis to vanillin (37). Biotechnological methods have also been employed, using microbial fermentation of substrates like ferulic acid to produce vanillin (38). Other examples include benzaldehyde, a compound with a characteristic almond flavor which is synthesized from toluene or benzyl chloride and ethyl maltol which has a sweet, candy-like flavor and is synthesized through the reaction of maltol with ethylating agents (39).

Flavor enhancers do not impart a specific flavor but intensify the taste of food by interacting with taste receptors. They are widely used in processed foods to enhance palatability. Some examples include monosodium glutamate (MSG), a sodium salt of glutamic acid, used to enhance savory (umami) flavors in a variety of dishes, disodium inosinate and disodium guanylate, often used in combination with MSG, to enhance umami taste (40, 41) and hydrolyzed vegetable protein (HVP), produced by breaking down proteins into amino acids, used to boost savory flavors in soups, sauces, and snack foods (42).

### 2.3 Benefits and risks of natural vs. artificial flavor additives

The debate between natural and artificial flavors is longstanding, often driven by consumer preferences and perceptions of health and safety (43). Natural flavors, derived from natural food sources, are perceived as superior and healthier by many consumers. On the other hand, artificial flavors, created through chemical processes, are scrutinized for their safety and potential health risks (44, 45).

Both natural and artificial food additives can trigger allergic and immunologic reactions in sensitive natural. These reactions can range from mild to severe and involve various mechanisms. Natural additives, such as menthol and peppermint oil, as well as artificial additives, such as MSG and certain sweeteners, have been associated with allergic responses (46, 175). The safety of food additives is also compromised by contaminants such as heavy metals and formaldehyde, which can be found in both natural and artificial additives (48).

However, scientific evidence does not always support this perception (45). As a matter of fact, natural additives can cause allergic reactions like or even more severe than artificial additives (49). Additionally, natural and artificial additives are chemically identical or similar. For example, vanillin can be extracted from vanilla beans or synthesized chemically, yet the molecular structure and potential health impacts are the same (50, 51).

Overall, the safety of food additives, whether natural or artificial, involves complex considerations of allergic reactions, potential contaminants, and misleading distinctions between “natural” and “artificial” labels. Proper regulatory measures and consumer awareness are essential to ensure the safe use of these substances in the food industry (44).

### 2.4 Health and safety considerations

Food additives are subjected to rigorous safety evaluations to ensure they do not pose risks to human health. The toxicity and safety levels are assessed through extensive studies, typically involving in vivo analyses, to determine potential adverse effects (52). One risk assessment tool is to identify the highest dose at which no adverse effects are observed, known as the No Observed Adverse Effect Level (NOAEL) (53). The NOAEL is derived from toxicological studies and is crucial for establishing safe exposure levels for humans. For example, when evaluating food additives such as glutamic acid, and its salts, neurodevelopmental toxicity studies revealed a NOAEL of 3,200 mg/kg of body weight per day. This NOAEL is used to calculate acceptable daily intake (ADI) by applying safety factors (54).

Food additives must meet stringent regulatory standards before they can be approved for use. Regulatory agencies such as the U.S. Food and Drug Administration (FDA) and the European Food Safety Authority (EFSA) play critical roles in this process. The FDA evaluates food additives through a comprehensive safety and risk assessment procedure, which includes reviewing toxicological data and determining risk thresholds acceptable exposure limits (55). The FDA uses a safety factor approach, typically applying a 100-fold safety factor to the NOAEL to account for variations (56). The EFSA has a different strategy, conducting thorough re-evaluations of food additives to ensure their safety. For instance, the re-evaluation of silicon dioxide (E551) concluded that while no significant toxic effects were tested at the highest doses, further characterization of particle size distribution was necessary to confirm safety (57). Table 2 summarizes the current food and feed additives legislation in different regions and organizations.

**Table 2:** Overview of current food additives legislation across regions and organizations.

Org.	Legislation	Description	Last modified
European Union (EU)	Reg (EC) No 178/2002	General requirements and principles of food legislation	2022
	Reg (EC) No 1333/2008	Harmonize, ensure safety, quality, and ease of storage and use	2024
	Reg (EU) No 231/2012	Sets specifications for food additives listed in Annexes II and III of Reg 1333/2008	2024
	Reg (EU) No 1169/2011	Specifies how additives must be declared on food	2018
	Reg (EC) No 1831/2003	Classifies feed additives according to categories	2003
	CR 257/2010	Sets up a program to evaluate additives approved before Reg No. 1333/2008	2010
	Reg (EC) No 1334/2008	Establishes rules for using “natural” in flavors and lists approved substances	2008
	Reg (EC) No 2065/2003	Outlines the process for evaluating and approving smoke flavorings	2021
	EFSA guidance	Provides oversight for food additive registration applications	Cont. updated
United States (US)	FD&C Act	Regulates food and color additive safety	2009
	FAA	Requires food additive safety premarket approval	2006
	CAA	Requiring pre-market approval of color additives used in food, drug, cosmetic, and certain medical devices	Cont. updated
	CFR	Contains rules for food additives (parts 170–180) and color additives (parts 70–82)	Cont. updated
Codex Alimentarius	GSFA	Provides a list of additives permitted for use in foods and their conditions of use	Cont. updated
Australia and New Zealand	FSC (1.3.1)	Covers food additives and specifies permitted uses in food	Cont. updated
	FSC (1.3.2)	Deals with vitamins and minerals	Cont. updated
Canada	Food and Drugs Act	Governs food additives and their use in food products	Cont. updated
	Permitted Food Additives	An official list of food additives that have been approved for use in Canada	Cont. updated
Japan	FSA	Introduction of a positive list system for food additives	2020
China	NFSS	Sets out permissible food additives and their conditions of use	Under revision

EFSA, European Food Safety Authority; FD&C Act, Federal Food, Drug, and Cosmetic Act; FAA, Food Additives Amendments; CAA, Color Additive Amendments; CFR, Code of Federal Regulations; GSFA, Codex General Standard for Food Additives; FSC, Food Standards Code; FSA, Food Sanitation Act; NFSS, National Food Safety Standard for Uses of Food Additives; CR, Commission Regulation. Legislative references were obtained was sourced from the official websites of relevant organizations including the European Commission (https://ec.europa.eu/food/safety_en), the United States FDA (https://www.fda.gov/), the Codex Alimentarius (http://www.fao.org/fao-who-codexalimentarius/en/), and regulatory authorities of Australia (https://www.foodstandards.gov.au/), New Zealand (https://www.mpi.govt.nz/), Canada (https://www.canada.ca/en/health-canada.html), Japan (https://www.mhlw.go.jp/english/), and China (http://www.nhc.gov.cn/), and from Barciela et al. (169).

Consumer perceptions of food additives significantly influence their acceptance. There is a growing concern among consumers about the potential health effects of food additives, particularly in vulnerable populations such as children (58). Ensuring transparency in the regulatory process and providing clear information about the safety and benefits of food additives can enhance consumer trust and acceptance (59). Regulatory bodies are encouraged to continuously update safety assessments and communicate findings effectively to the public (60).

## 3 Traditional methods of flavor production through SSF

SSF is a traditional method used in the production of various fermented foods in different cultures (61). This process involves the growth of microorganisms on solid materials in the absence of free-flowing water, resulting in unique flavors and textures in the final products. SSF processes are inherently complex, involving dynamic interactions between solid, liquid, and gas phases, as well as intricate mass and heat transfer mechanisms. While traditional SSF methods have been widely used for centuries, many are still based on empirical knowledge rather than rigorous scientific engineering principles (62, 63). However, recent advances in SSF engineering have focused on raw material pretreatment, process parameter detection, and equipment innovation to meet the demands of smart manufacturing and sustainable production (62). Despite these efforts, one of the major challenges in SSF remains the limited understanding of microbial interactions and their impact on fermentation dynamics. This knowledge gap hinders precise control of fermentation outcomes and requires integrated study approaches that combine microbiological and engineering perspectives to identify key variables that affect product yield and quality (62, 64).

The core microbiota in traditional SSF includes specific microorganisms that are critical for the production of metabolites that determine the sensory quality of the final product. For instance, in the production of Chinese Maotai-flavor liquor, the core microbiota includes genera such as *Pichia*, *Schizosaccharomyces, Saccharomyces, Zygosaccharomyces*, and *Lactobacillus*. These microorganisms are involved in the conversion of alcohol to acids, which is essential for flavor development (15, 65). In the case of Chinese cereal vinegar, the metabolic pathways of organic acids such as acetic and lactic acids are regulated by environmental factors like temperature and the presence of acetic acid. The dominant microorganisms in this process are *Lactobacillus* and *Acetobacter*, which contribute significantly to the flavor profile (15).

The traditional methods of SSF, while effective, pose challenges related to food safety and quality control (66). There is a growing need to integrate food safety management systems to address concerns such as the accumulation of toxic compounds and to improve the overall safety and quality of fermented foods (67). For example, the SSF process involves complex interactions between various microorganisms, which can pose safety risks if not properly managed. The lack of built-in safeguards against undesirable microbial growth and toxins is a significant challenge (61). Other concerns for small-scale productions are the improper use of chemicals, such as pesticides and antibiotics, and inadequate processing and storage, which can result in the accumulation of toxic compounds like mycotoxins and biogenic amines and the lack of ingredient quality (67).

Despite all this, fermented plant-based foods remain as one of the safest foods in the world to consume as fermenting vegetables not only allows extending the shelf life of food immensely, but also brings other benefits, including inhibiting the growth of pathogenic microorganisms (68).

## 4 Smf approaches to flavor production

In SmF, microorganisms grow in nutrient-rich media, producing a variety of VOCs. This method is commonly used for umami-enhancing nucleotides, such as inosinate and disodium guanylate, which are derived from yeast or bacterial fermentation (70). Similarly, further biotransformation can convert precursor molecules into complex flavors, such as microbial conversion of ferulic acid into vanillin, offering a cost-effective alternative to vanilla extraction (71, 72).

Various microbial strains are able to produce key aroma compounds under liquid conditions such as esters (fruity), aldehydes (nutty and floral), ketones (buttery), and alcohols (rose-like scents). Esters, responsible for fruity and floral notes, are commonly produced by *S. cerevisiae*, *Lactococcus lactis*, and *Kluyveromyces marxianus* through the interaction of carboxylic acids and alcohols (73, 177). Aldehydes, such as benzaldehyde (almond aroma) and vanillin, are formed via microbial oxidation and reduction pathways (69). Ketones, particularly methyl ketones, contribute to buttery and cheesy flavors. For instance, *Penicillium roqueforti*, used in blue cheese production, generates methyl ketones through the decarboxylation of fatty acids, resulting in a characteristic aged aroma (74). Alcohols, such as 2-phenylethanol, provide floral and rose-like scents and are commonly produced by *K. marxianus* and *Saccharomyces cerevisiae* via amino acid metabolism (71, 74). SmF is also capable of synthesizing terpenes, which contribute to citrus, herbal, and resinous aromas (75). Engineered *E. coli* strains have been used to biotransform limonene, a key citrus compound, highlighting the potential for microbial production of essential oil components (76, 77). Additionally, pyrazines, which provide roasted, nutty, and cocoa-like flavors, can be produced by *Bacillus subtilis* and *Corynebacterium glutamicum* through fermentation processes involving amino acid precursors (78).

SmF remains a valuable tool for scalable and controlled flavor production, particularly for industries requiring consistent and high-yield aroma compounds. However, SSF is gaining traction due to its sustainability and efficiency in utilizing agricultural waste as a substrate.

## 5 SSF approaches to flavor production

The technique of SSF has attracted attention as a promising method for enhancing flavor production. SSF is a type of fermentation that occurs in the absence of free water (79). This process makes it possible to use significant amounts of organic material as substrates without preparation, creating an environment that mimics nature and allows microorganisms to grow. This approach shows promise, as it involves extracting natural tastes and aromas from microorganisms. This approach requires creating a complex growth medium. This closely resembles the organisms’ natural environment (80). SSF is distinguished from the more widely used SmF by the fact that it involves fermentation on solid particles in the absence of free water (12).

**Figure 1:**
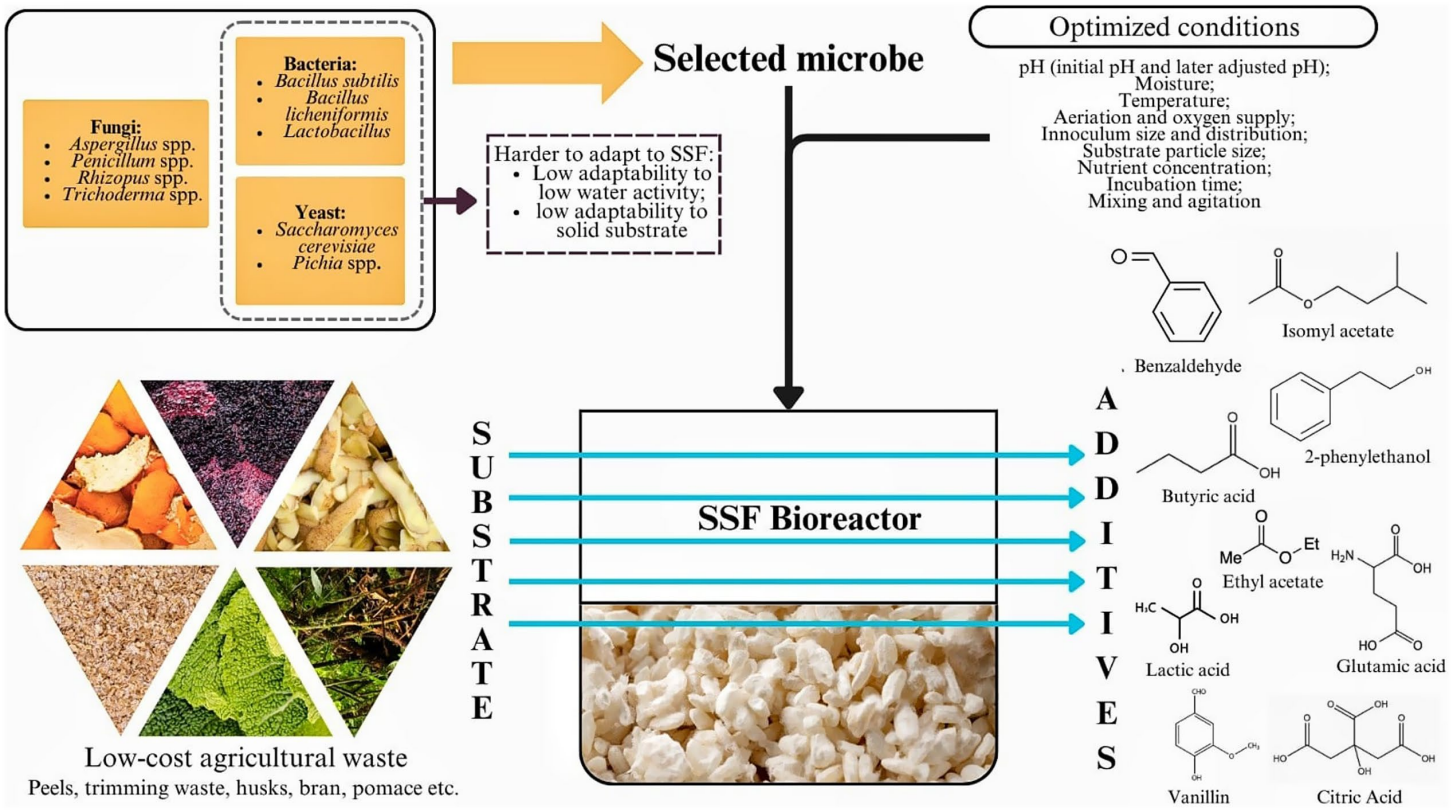
Solid-state fermentation (SSF) process utilizing low-cost agricultural waste as a substrate. Created with Canva.com.

As previously mentioned, consumers are increasingly seeking clean-label ingredients and favoring fermentation-derived flavors over synthetic additives. SSF aligns with this trend, utilizing natural microbial processes to enhance taste, aroma, and nutritional value, and reducing reliance on artificial flavor enhancers (11). Furthermore, the global shift toward sustainability has increased interest in fermentation methods that repurpose agricultural waste, establishing SSF as a circular economy innovation in food production. SSF’s ability to produce exotic and complex flavors also appeals to the rising demand for premium, artisanal, and plant-based food products, further driving market adoption (81).

SSF has been shown to be able to manufacture aromatic compounds from agricultural and industrial waste such as sugarcane bagasse, coconut husks, and coffee pulp (82). This method reuses the abundant and often underutilized agricultural residues to produce valuable bioproducts, contributing to environmental conservation and economic benefits. This section will explore the various characteristics, advantages, and limitations of SSF to produce food additives using agricultural waste Figure 1.

### 5.1 Advantages and limitations of SSF over SmF

**Figure 2:**
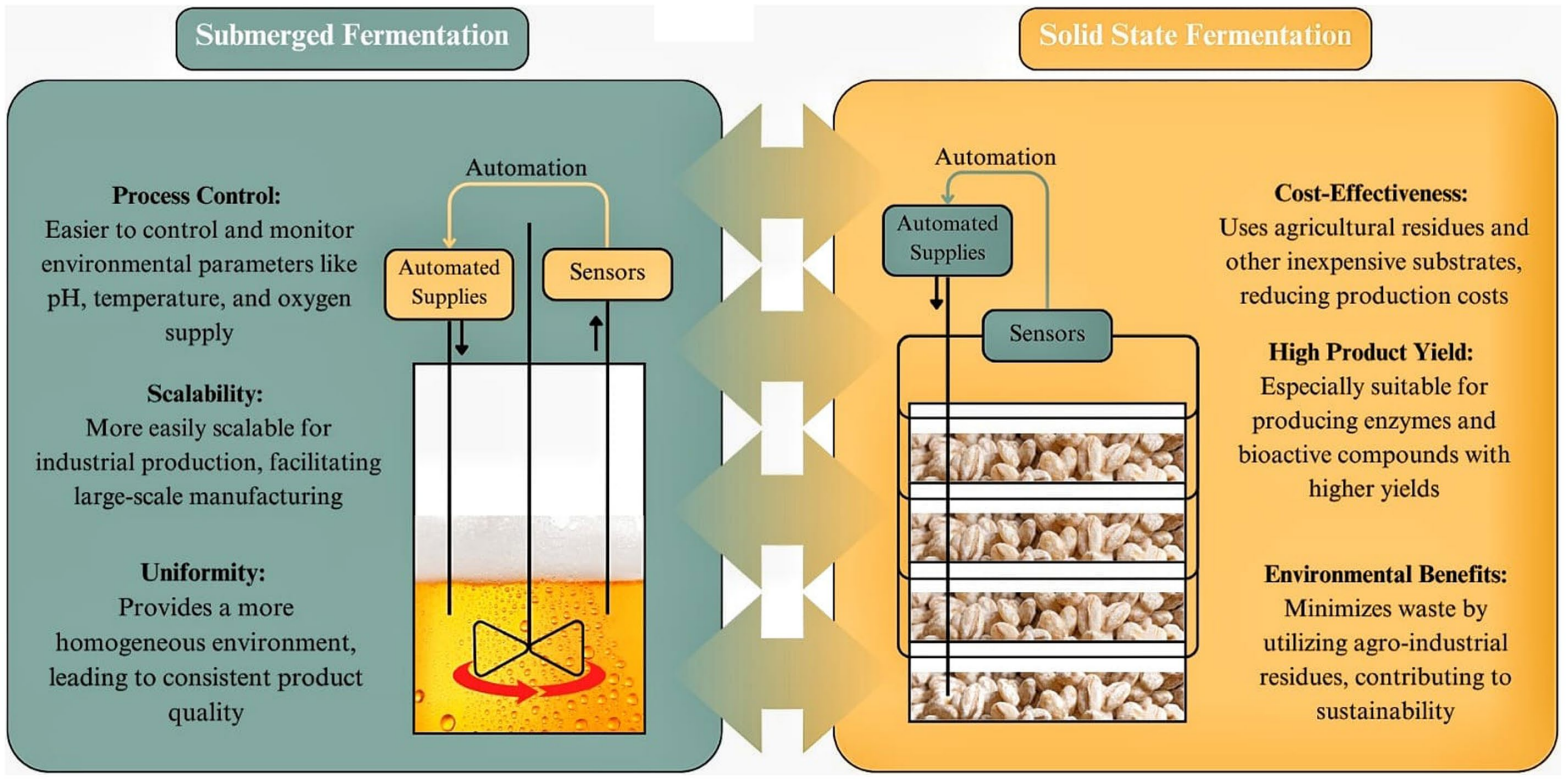
SSF vs. SmF” (Solid-State Fermentation vs. Submerged Fermentation). Created with Canva.com.

SSF offers several advantages over SmF, particularly in specific applications such as enzyme production, bioproducts, and nutraceuticals. The unique characteristics of SSF, including its ability to operate in low-water environments and utilize solid substrates, contribute to enhanced productivity, efficiency, and sustainability in industrial fermentation processes (83). The main differences between SSF and SmF are illustrated on Figure 2. One of the key advantages of SSF is its higher productivity, particularly in enzyme production. Studies have shown that enzymes such as invertase, pectinases, and tannases exhibit greater yields and higher specific growth rates in SSF compared to SmF (84, 85). This is attributed to the natural adaptation of many microorganisms, particularly fungi, to grow on solid matrices, leading to improved metabolic activity and enzyme secretion. Additionally, SSF is characterized by lower water and energy requirements, making it a more environmentally friendly alternative to SmF (84). The reduced need for water not only lowers operational costs but also minimizes wastewater generation, which is a significant challenge in large-scale SmF systems. Furthermore, SSF products tend to exhibit higher stability and concentration, which can facilitate storage and transportation while reducing the need for extensive downstream processing (86).

Another major advantage of SSF is the ability to utilize a wide range of solid substrates, including agricultural and industrial waste materials. This feature not only reduces raw material costs but also promotes waste valorization and circular economy principles, further enhancing the sustainability of SSF-based processes. The low water activity in SSF also leads to reduced catabolic repression, allowing for the efficient production of specific metabolites and enzymes that may be inhibited in SmF due to excess water and nutrient dilution (84). A further advantage of SSF is its lower sterility requirements. The limited availability of free water in SSF reduces the risk of microbial contamination, thereby decreasing the need for stringent sterilization procedures and further simplifying production (87). This advantage is particularly relevant for large-scale fermentation operations, where sterility maintenance is a major cost factor. Lastly, SSF offers the potential for mixed microbial cultivation, allowing for the simultaneous growth of different microbial species, particularly fungi, which specialize in breaking down water-insoluble substrates (84). This capability can enhance the diversity and yield of bioactive compounds, further broadening the application of SSF in the production of enzymes, bioactive metabolites, and natural flavors (88).

### 5.2 Process parameters and optimization

Factors, such as different strains and environmental conditions, can significantly affect the range of flavor compounds found in fruits and flowers. Table 3 provides a detailed overview of SSF parameters, requirements, effects, and other important aspects.

**Table 3:** Overview of SSF parameters, their requirements, and effects.

Parameter	Description	Requirements	Effects	Ref.
Moisture	Crucial for microbial development	40–60% for fungi, up to 85% for bacteria	Limits solubility and microbial growth Reduces porosity and oxygen delivery Limits high humidity, particle agglomeration and transport	Singhania et al. (89), Martins et al. (90), Orzua et al. (91), Sadh et al. (92)
pH	Affects volatiles and flavor	Microbe and substrate specific	Acidic: enhances volatiles and alkaline solubilizes waste	Bolaji and Dionisi (96), Cheah et al. (97)
Temperature	Influences growth and enzyme production	20–55°C for fungi, varying for bacteria	Decreases microbial activity and production High temperature may increase enzyme production	Jiang (26), Pandey (100), Abraham et al. (101)
Inoculum and nutrients	Microbes selected based on substrate and objective	Filamentous fungi are optimal	Affects microbial growth and production Adequate concentration is key to not limit growth	Cai et al. (103), Juanssilfero et al. (105), Carrau et al. (107)
Initial fermentation	Importance of inoculum concentration	Influences microbial growth	Growth limited by inadequate inoculation Mass transfer limited by excessive inoculation	Juanssilfero et al. (105), Krishna and Nokes (170)
Enzymatic activity	Effect of temperature on enzyme production	Depends on type of microorganism	Temperature-sensitive enzyme activity	Yazid et al. (13), Abraham et al. (101)

Moisture content plays a crucial role in SSF as different microorganisms require specific hydration levels for optimal growth. Fungi typically thrive at 40–60% moisture (89), while bacteria may require up to 85% (90). Maintaining appropriate moisture levels is essential for nutrient availability, oxygen diffusion, and carbon dioxide (CO_2_) exchange, all of which influence fermentation efficiency (91). Excess moisture can reduce porosity, weaken substrate structure, and restrict oxygen diffusion, creating an environment unfavorable for microbial activity. Conversely, low moisture levels limit nutrient solubility, inhibiting microbial growth, and enzyme production (92). For bacterial cultures, moisture content directly affects microbial development and alters the physicochemical properties of solid substrates, impacting overall productivity (11). Additionally, high humidity levels can cause particle agglomeration, restrict gas exchange, and increase microbial competition (93). The moisture range in SSF typically varies between 30 and 85%, depending on the substrate and microbial strain used (94).

pH plays a critical role in flavor development during SSF, as it directly affects the production of volatile compounds and microbial metabolism (95). Specific pH levels influence the formation of distinct aroma compounds, while fermentation efficiency can also be enhanced through pH regulation. For instance, the use of pH buffers has been shown to increase ethanol output (96). Acidic conditions typically enhance VOC production, whereas alkaline environments facilitate volatile generation by solubilizing organic waste (97). Additionally, microbial diversity influences pH fluctuations during fermentation, impacting flavor profiles. For example, Lu et al. (98), used potato peel fermentation at pH 7 to achieve specific flavor characteristics, highlighting the importance of optimizing pH conditions for desired sensory outcomes.

Temperature is another key parameter affecting microbial activity and product synthesis in SSF. An increase in temperature during fermentation signals microbial growth, particularly in aerobic conditions, where oxygen supply and CO_2_ exchange generate metabolic heat (99). However, excessive heat can negatively impact microbial survival and enzyme activity, reducing fermentation efficiency (100). In some cases, higher temperatures enhance enzyme production, improving substrate conversion and aroma development (13, 101). Fungal strains in SSF typically thrive between 20°C and 55°C, with optimal conditions varying by species and target metabolites. Many fermentation processes rely on mesophilic microbes, which tolerate temperatures up to 50°C (102). Additionally, metabolic heat is generated during microbial proliferation, influencing the overall SSF process (178).

Inoculum selection and nutrient supplementation are also critical factors in SSF success. The choice of microorganism depends on substrate compatibility and desired flavor outcome (79). While yeast, bacteria, and fungi have all been explored for SSF, filamentous fungi are particularly advantageous due to their ability to grow under low-water activity conditions (103). However, maintaining the correct physiological conditions is essential for secondary metabolite production. For example, Crafack et al. (104), reported that when the inoculum responsible for flavor compound synthesis fails to maintain proper physiological balance, production efficiency declines.

Early fermentation determines the subsequent course of the culture. In the fermentation process, the size of the inoculum determines the development of the microorganisms, since an inadequate inoculum concentration is insufficient to initiate microbial growth, and a large inoculum concentration restricts mass transfer (66, 105). In SSF, inoculum concentration is an important metric (106). Sporulation is influenced by metabolic effects such as carbon, nitrogen, minerals and vitamins. Carbon provides energy for the development of microorganisms, while glucose, starch, cellulose, maltose, lactose and glycerol are sources of carbon (107). Ammonium tartrates, amino acids, sulfates, nitrate, sodium nitrate, peptones, and urea are all nitrogen sources (80).

### 5.3 Large-scale adaptation of SSF

While SSF offers numerous advantages, its large-scale industrial deployment remains challenging due to reactor design limitations, process scalability, and operational constraints (82). Unlike SmF, which can be easily adapted to traditional stirred-tank bioreactors, SSF requires specialized reactor configurations that accommodate solid substrates while ensuring adequate oxygen transfer, heat dissipation, and microbial stability. The heterogeneous nature of SSF substrates complicates reactor engineering, often leading to issues with mass and heat transfer that can affect productivity on an industrial scale (64, 108).

**Figure 3:**
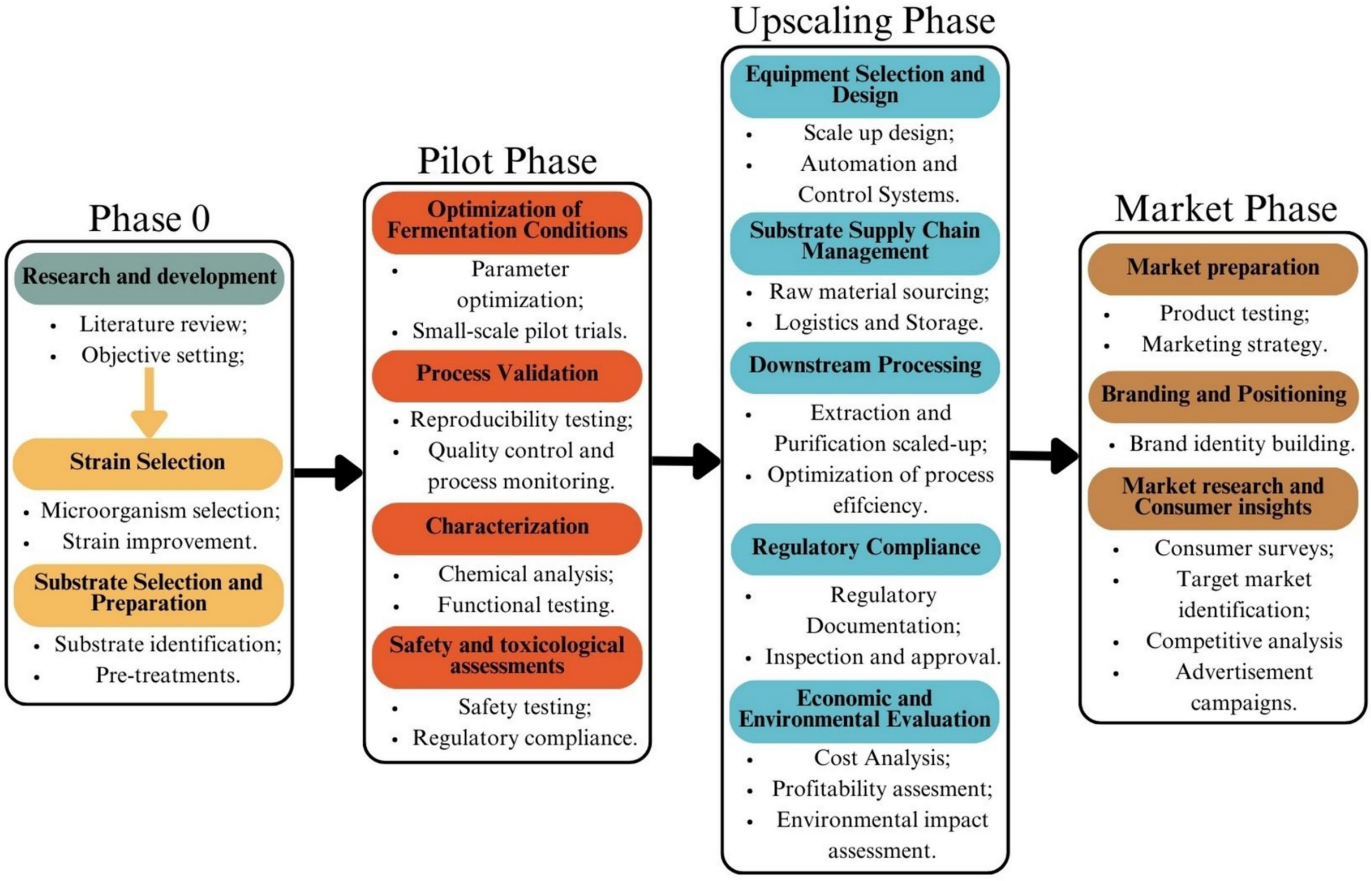
Process of producing a new food additive via SSF. Created with Canva.com.

One of the key steps in scaling up SSF is pilot-scale optimization, where small-scale laboratory processes are tested in intermediate-sized reactors to assess feasibility before full-scale production. Several factors must be optimized in these pilot stages, including moisture control, aeration, and substrate particle size, as these directly impact microbial activity and product yield (109). Unlike SmF, which operates in a homogeneous liquid environment, SSF reactors must maintain a delicate balance between substrate porosity and microbial access to nutrients. Large-scale SSF reactors, such as tray, packed-bed, and rotating drum bioreactors, are being developed to address these challenges, but further improvements are needed to enhance productivity and cost-efficiency. Another major consideration for large-scale SSF adoption is contamination control. While SSF’s low water activity naturally reduces bacterial contamination, maintaining sterile conditions in high-volume production remains a challenge, particularly in open-system fermentations. In contrast, SmF requires highly pure analytical-grade media, increasing production costs and making it less sustainable in terms of raw material utilization and waste generation (108). The ability of SSF to leverage agricultural and food processing waste as substrates makes it a more environmentally friendly approach, promoting circular economy principles (110, 111). The optimization process, including the different scales the process passes through is illustrated on Figure 3.

### 5.4 Vegetable wastes as a sustainable substrate for SSF

Agriculture generates vast amounts of fresh food for consumers and raw materials for the food processing industry (112). However, not all agricultural output is suitable for food and industrial use. Agro-industrial residues, which include harvesting by-products, are a significant component of agricultural waste (92). Each year, approximately 5 billion metric tons of agricultural waste are produced worldwide, with an estimated 1.3 billion tons of food, primarily fruits and vegetables, going to waste (113). Much of this waste is discarded, burned, or buried, resulting in environmental contamination, although some of it is reused as animal feed or bedding (114). The use of agro-industrial waste in SSF presents a unique opportunity for bioconversion into valuable products. Agro-industrial residues such as sawdust, fruit peels, and other plant-based waste are particularly well-suited for SSF due to their abundance, low cost, and chemical composition. These substrates contain cellulosic, hemicellulosic, lignocellulosic, sugar, and protein contents, ranging from 15 to 50%, making them ideal nutrient sources for microbial metabolism (92). Substrate preparation is essential, as breaking down waste into smaller particles facilitates mycelial penetration and colonization, providing both carbon and energy sources for microbial growth (115). Additionally, the solid matrix in SSF acts as both a nutrient source and a support system for microbial adherence (116). Examples of SSF applied to agricultural waste for flavor production can be found on Table 4.

**Table 4:** Flavor creation through the use of microorganisms in the SSF by-products of agricultural and food wastes.

Microorganism	Agricultural waste	Flavors produced	Products	Ref.
*Sphingobium* sp.	Citrus peel	Lilacs or linden, and blossoms	α-terpineol	Molina et al. (167)
*Saccharomyces cerevisiae*	Orange Peel	Fruity	Ester	Mantzouridou et al. (120)
*Hanseniaspora valbyensis; Saccharomyces cerevisiae; Hanseniaspora uvarum*	Apple pomace	Fruity, green apple, grass, almond, sweet, creamy, vanilla, warm, spicy, lemony, and citrusy	Acetaldehyde, benzaldehyde, vanillin, cinnamaldehyde, and citral	Madrera et al. (171)
*Enterobacter hormaechei*	Banana peels	Vanillin	Biovanillin	Saeed et al. (142)
*Kluyveromyces marxianus; Debaryomyces hansenii*	Tomato pomace, and red pepper pomace	Fruity, grape, ethereal, sweet, and rum-like notes	Aldehydes-1, alcohols-3, and esters-7	Güneşer et al. (150)
*Pichia kudriavzevii*	Sugarcane bagasse	Rose	2-phenylethanol	Martínez-Ávila et al. (165)
*Saccharomyces cerevisiae; Kluyveromyces marxianus*	Potatoes and oranges, municipal solid residue, brewer’s spent grain, molasses, and cheese whey	Fresh, woody, and pine-like aroma	ε-pinene	Aggelopoulos et al. (12)
*Yarrowia lipolytica*	Non-hydroxylated fatty acids	Fruity, milky, coconut	γ-Dodecalactone and δ-Decalactone	Marella et al. (164)
*Ceratocvstis fimbriate*	Cassava bagasse, amaranth, soya bean, and apple pomace	Fruity aroma	Aldehyde-1, alcohols-6, esters-5, ketones-2, acid-1	Bramorski et al. (172)
*Kluyveromyces marxianus*	Palm Bran, cassava bagasse	Fruity Aroma	Ethanol and acetaldehyde ethyl acetate	Medeiros et al. (186)
*Saccharomyces cerevisiae*	Beet peels	Malty and fruity	Acetate esters, and hydroxybenzoic acids	Correia-Lima et al. (138)
*Trichoderma harzianum*	Sugarcane bagasse	Sweet and caramel-like odor	6-pentyl-α-pyrone	Ladeira et al. (156)

The choice of substrate significantly influences yield and efficiency in flavor production. Organic waste from the agricultural sector offers an ideal fermentation medium due to its abundant supply, cost-effectiveness, and rich chemical composition (11). By valorizing waste as a substrate, SSF contributes to waste reduction and environmental sustainability. Several factors affect substrate selection, including sugar content, precursor availability, and porosity, all of which influence microbial growth and flavor compound formation. For example, apple peel has been used as a substrate to produce 2-phenylethanol, with optimized substrate compositions incorporating nutrients such as MgSO_4_, MnSO_4_, and FeSO_4_, enhancing production efficiency (117). Particle size is another critical factor in SSF. While small particles offer a greater surface area for microbial adhesion, they may also lead to substrate agglomeration, reducing oxygen diffusion and hindering microbial growth (118). Conversely, larger particles improve aeration efficiency but reduce microbial attachment sites (100). An ideal SSF substrate must contain essential nutrients and may require additional supplementation to support microbial activity (66). Waste materials can be upcycled or valorized into substrates for fermentation, transforming what was once discarded into a valuable resource for aroma production (119). Sterilization is also a crucial consideration, as removing contaminants enhances yeast growth and flavor synthesis. For instance, sterilized orange peel waste has shown improved fermentation efficiency and volatile compound production (120). Thus, carefully optimizing fermentation parameters can increase both the quantity and diversity of flavors derived from agricultural waste (76).

## 6 Microorganisms in SSF

Microorganisms play a fundamental role in SSF, enabling the production of bioactive compounds and valuable flavor molecules. Bacteria, filamentous fungi, and yeasts are the primary microbial groups involved in SSF, each contributing distinct metabolic activities that influence the fermentation process. While unicellular bacteria and yeasts typically form biofilms, filamentous fungi develop intricate mycelial networks that penetrate and colonize solid substrates (121). The structure of these microbial communities impacts nutrient diffusion, moisture retention, and oxygen availability, all of which are critical for optimal fermentation performance (122). Depending on the desired fermentation outcome, microorganisms can be used individually or in co-cultures to enhance metabolic diversity and improve product yields (179).

### 6.1 Microbial metabolic pathways in flavor formation

Microorganisms contribute to flavor development through various metabolic pathways. The production of flavor compounds in SSF is driven by the diverse metabolic activities of microorganisms, including bacteria, yeasts, and filamentous fungi (121). Key metabolic pathways involved in flavor formation include proteolysis, lipolysis, carbohydrate metabolism, and amino acid catabolism, each playing a distinct role in generating esters, alcohols, aldehydes, and organic acids. Understanding these metabolic pathways is essential for optimizing fermentation conditions, microbial selection, and substrate composition to improve the yield and quality of natural flavors (123).

#### 6.1.1 Protein metabolism and amino acid degradation

*B. subtilis* is a well-documented producer of stable serine proteases and alkaline proteases, which play a crucial role in the breakdown of proteins during fermentation (124). These enzymes are essential in the hydrolysis of complex proteins into smaller peptides and free amino acids, which serve as precursors for flavor compounds. The activity of alkaline proteases is particularly significant in high-pH environments, where they enhance the formation of umami-rich peptides, contributing to the depth of flavor in fermented foods. In addition to *B. subtilis*, fungi such as *Rhizopus, Mucor*, and *Aspergillus oryzae* are known for their ability to produce highly active acid proteases, which function optimally in low-pH conditions (125). These enzymes are widely used in food fermentation processes, where they degrade proteins into smaller peptides, free amino acids, and aroma precursors. This enzymatic breakdown not only contributes to the development of savory and complex flavors but also influences the texture and mouthfeel of fermented products (126).

Ammonia is decarboxylated and dehydrogenated to form aromatic compounds such as ketones, aldehydes, acids, alcohols, phenols, and indoles (127). Bacteria can metabolize amino acids in two ways. Amino acid lyase degrades amino acid chains into phenol, indole, and methyl mercaptan, found in yeast, *Micrococcus*, and *Brevibacterium*. Microbial metabolism generates taste by transamination, regulating the metabolism of aromatic amino acids like such as tryptophan, tyrosine, and methionine (128). The amino transferase enzyme converts α-ketoic acid to alcohols, aldehydes, ketones, and carboxylic acids, facilitating amino acid metabolism (129). Isoleucine, valine, and leucine, three branched amino acids, can produce fruity and malty flavors via compounds such as 2-methyl-butyral and 3-methyl-butyral. 3-methyl butyral has a fruity flavor, whereas 2-methyl butyral has a malty taste (130, 131). *Wickerhamomyces* and *Clostridium* are the primary bacteria involved in amino acid metabolism, affecting the flavor of cheese, wine, and sausages. By breaking down amino acids such as glycine, glutamic acid, alanine, and leucine, these bacteria can produce flavor compounds (132).

#### 6.1.2 Lipid metabolism and fatty acid breakdown

Lipids function as solvents for aromatic components, influencing scent via biological processes. Fermented meats generate taste molecules via lipid oxidation (133). Endogenous enzymes and microorganism-produced lipases, such as *Bacillus, Pseudomonas, Candida*, and others, promote lipid breakdown by autooxidation and enzymatic oxidation (189). Lipase degrades lipids, releasing fatty acids. β-oxidation produces taste compounds including aldehydes, ketones, and alcohols. Lipid oxidation generates hydroperoxides via a complicated chain reaction involving oxygen (190). Hydroperoxides’ instability leads to the formation of free fatty acids, which can then transform into hydrocarbons, alcohols, aldehydes, ketones, acids, and other compounds by numerous routes (Ayala et al., 2014). *Staphylococcus equine* or *Staphylococcus xylose* can degrade fatty acids in sour meat with low salt, producing linoleic acid, octadecenoic acid, palmitic acid, and other fatty acids. These acids are then further oxidized and degraded to produce linear aldehydes like nonanal and 2-heptanal, contributing to the distinct flavors of fermented sour meat, including cheesy, fruity, and sweet notes (180).

#### 6.1.3 Carbohydrate metabolism and nucleotide contribution to flavor

Carbohydrate metabolism impacts food flavor (134). Major pathways include glycolysis, the pentose phosphate pathway, and the tricarboxylic acid (TCA) cycle. Microbial carbohydrate metabolism produces pyruvate, a key taste intermediary, which lowers the food’s pH, inhibits bacteria growth, and gives it a sour taste. Pyruvate converts into flavor molecules via metabolic cycles such as the TCA cycle. LAB convert carbs into lactic acid, amino acids, organic acids, and polysaccharides, affecting horse milk wine’s properties (135). 5′-nucleotides enhance the umami flavor of fish products and are obtained by breaking down adenosine triphosphate (ATP) and free fatty acids (181). The productin of flavor-related nucleotides (adenosine monophosphate [AMP], inosine monophosphate [IMP], and guanosine monophosphate [GMP]) is associated with microbial metabolic activity. Microorganisms employ phosphodiesterase to convert nucleic acids to nucleotides. Nucleotides combine with amino acids to enhance umami flavor and contribute to the Maillard reaction for tastier chemicals. *Lactobacillus* and *Staphylococcus* ferment the sour meat with minimal salt, reducing IMP and creating hypoxanthine for a bitter taste (180).

#### 6.1.4 *De Novo* synthesis and biotransformation

*De Novo* synthesis involves a comprehensive metabolic process, where proteins, lipids, and carbohydrate catabolism contribute to the formation of primary metabolites. These metabolites are then transformed into a mixture of aromatic compounds, which are key to flavor production (182). When agricultural waste or by-products are used as substrates, sugar supplementation is often required to stimulate microbial growth in the early stages. However, excessive sugar can cause catabolite suppression, limiting further metabolic activity. An alternative approach to overcome nutrient limitations in SSF is the combination of diverse waste substrates, creating a self-sustaining fermentation medium that does not require additional nutrients (12). A crucial strategy in *de novo* synthesis is the addition of metabolic precursors, which can induce the synthesis of specific flavors. For example, incorporating leucine into agri-food waste fermentation enhances isoamyl acetate production, a compound with a characteristic banana-like scent (136, 174). Optimization of fermentation conditions, such as nitrogen restriction and temperature control, has been demonstrated to enhance fruit fragrance synthesis using SCB and sugar beetroot molasses (137).

The potential of biotechnological methods in ester production has also been explored, with studies demonstrating the synthesis of esters from vegetable waste through SSF of orange peel with yeast cells (120). Similarly, beetroot peels, a common culinary waste, have been investigated as a brewing additive in beer production (138). Moreover, the optimization of *Bacillus licheniformis* MSJM5 fermentation conditions has proven effective for biovanillin synthesis, highlighting the application of biotechnology in transforming vegetable waste into valuable flavor compounds (139). In addition, in cases where fermentation has been used, there is usually a need for a bioseparation process that includes extraction, purification and chemical recovery. Due to the volatility and limited solubility of many flavor components, their recovery remains a challenge (69). Therefore, a thorough understanding of the properties of the target compound is necessary to select the most efficient extraction method to optimize selectivity and recovery from SSF systems.

**Figure 4:**
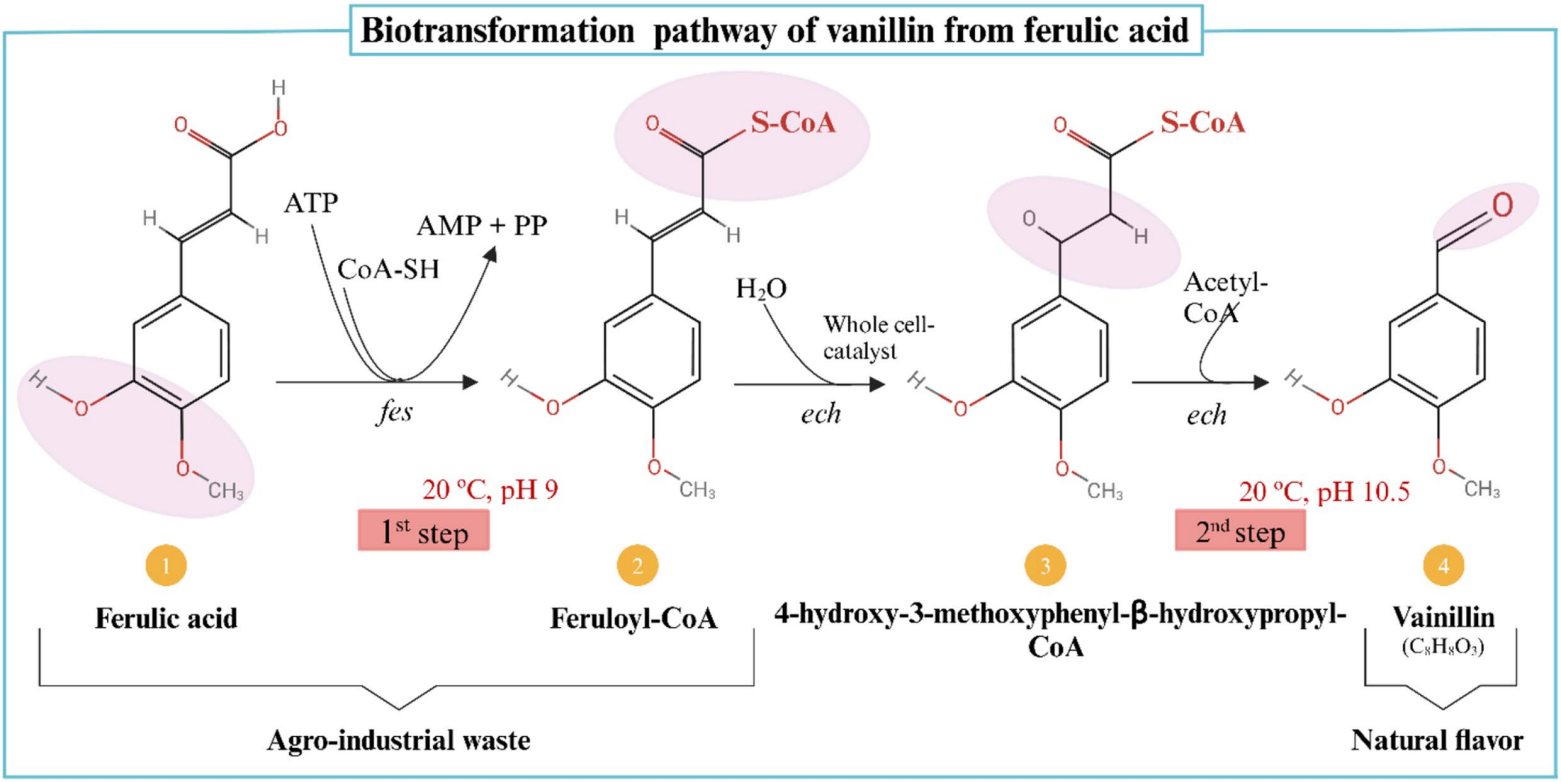
Ferulic acid is converted to vanillin by the enzymes feruloyl-CoA synthetase (fcs) and enoyl-CoA hydratase/aldolase (ech) (162, 188). Created with BioRender.com.

Biotransformation, on the other hand, refers to the microbial conversion of precursor compounds into desired flavor molecules, such as the conversion of ferulic acid into vanillin (182). Vanillin, a naturally occurring aromatic aldehyde in *Vanilla planifolia* pods, has wide applications in the cosmetic, pharmaceutical, and medical industries. To increase vanillin yield while reducing costs, biotransformation utilizes low-cost agricultural substrates (140). In a study on ferulic acid biotransformation, researchers demonstrated that *Amycolatopsis* sp. could increase vanillin content to 9.2 g after 32 h by supplementing 100 mg of vanillic acid during the fermentation process (183). This optimized concentration was significantly higher than the 212 mg vanillin achieved in fed-batch studies without supplementation. Ferulic acid levels in maize hulls, barley flour, maize bran, and sugar beets have been reported to vary significantly, influencing their suitability for bio-vanillin production (141). Additionally, ferulic acid concentrations in banana, pomegranate, and orange peel by-products range from 0.339 mg/g to 1.55 mg/g, reinforcing their potential use as fermentation substrates (182). Since ferulic acid is a key precursor in bio-vanillin synthesis, both natural and genetically engineered bacteria have been explored for fermentation optimization (142). While biotransformation efficiently yields single-aroma compounds, its commercial viability depends on optimizing fermentation parameters. Furthermore, consumer perceptions of genetically modified organisms (GMOs) present an additional challenge, as many do not regard GMOs as entirely “natural” (143). The biotransformation of ferulic acid into vanillin is illustrated in Figure 4.

The use of agricultural waste as a fermentation substrate has been widely explored for ferulic acid-based bio-vanillin synthesis, with substrates such as sugar beet pulp, rice bran oil, and fruit and vegetable by-products demonstrating strong potential. A central composite design approach has been used to optimize key fermentation parameters, achieving higher bio-vanillin yields of 0.476 g/100 g, compared to 0.029 g/100 g with unoptimized conditions (185). Additionally, engineered microorganisms hold promise in biotransformation applications, as they can be genetically modified to express specific enzymes required for efficient flavor production (76).

Overall, *de novo* synthesis and biotransformation offer powerful tools for the sustainable production of flavor compounds, utilizing low-cost agricultural waste to generate high-value aromatic molecules. The integration of advanced biotechnological strategies and process optimization will be essential to improving the efficiency, yield, and commercial viability of these sustainable flavor production methods (76, 144).

### 6.2 Lactic acid bacteria (LAB) in SSF and flavor enhancement

LAB are Gram-positive, non-spore-forming microorganisms primarily known for their ability to produce lactic acid, a defining feature of their metabolism. LAB play a crucial role in the fermentation of dairy, meat, vegetable, and fruit-based products, significantly influencing their sensory and textural properties. In addition to their role in fermentation, LAB exhibit probiotic properties, and contribute with antibacterial and antioxidant activities that enhance food safety and nutritional benefits (145). LAB’s enzymatic portfolio includes capabilities in aldehyde catabolism, ester production and hydrolysis, phenolic acid degradation, lipolysis, proteolysis, and peptide lysis, all of which contribute to the development of complex flavors and aromas in fermented foods (146). These metabolic activities transform proteins and lipids into free fatty acids, amino acids, and volatile aroma compounds, enhancing the overall flavor profile of food products.

LAB can be classified into homofermentative and heterofermentative species based on their fermentation pathways (147). Homofermentative LAB primarily convert glucose into two molecules of lactic acid, whereas heterofermentative LAB metabolize glucose into lactic acid, ethanol, and carbon dioxide (145). These fermentation mechanisms influence not only the acidity and preservation of food but also the formation of key aroma compounds that contribute to the characteristic flavors of fermented products (148).

LAB serve as starter cultures in numerous fermentation processes, with key genera including *Lactobacillus, Pediococcus, Streptococcus, Bifidobacterium*, and *Leuconostoc*. These bacteria are widely employed in the production of fermented dairy products, fermented meats, fruit juices, and vegetables, where they contribute to microbial stability, pH regulation, and the development of distinctive flavors (149). Research has demonstrated the impact of LAB on flavor modification in various fermented products. Studies by Güneşer et al. (150) and Spaggiari et al. (151) have examined LAB-driven flavor changes in fruit juices, dairy products, and meat fermentations, highlighting their role in the sensory enhancement of food. LAB fermentation influences substrate utilization, microbial strain selection, and fermentation modes, all of which affect the yield, purity, and complexity of the final product (152).

Additionally, the metabolic activity of LAB contributes to the production of aromatic compounds and their precursors, influencing the final sensory perception of fermented foods. This process can be strategically applied to enhance desirable aromatic notes or eliminate unwanted flavors, making LAB a crucial component in fermentation-based food processing (153).

### 6.3 Microbial and substrate interactions in flavor formation during SSF

The chemical and biological composition of vegetable waste plays a fundamental role in flavor compound formation during SSF. Various microbial species, including fungi, yeasts, and bacteria such as *Aspergillus* sp., *B. subtilis*, *Neurospora* sp., *Ceratocystis fimbriata*, and *K. marxianus*, have been identified as effective producers of aroma compounds (154). The selection of specific yeast strains can further refine the production of desired fragrance components. For example, *H. valbyensis*, *S. cerevisiae*, and *H. uvarum* have been found to produce fatty acids and associated ethyl esters when grown on apple pomace (177).

The composition of the substrate directly influences volatile compound synthesis. Studies have demonstrated that *Hanseniaspora* sp. can increase acetic ester production, while *S. cerevisiae* and *K. marxianus* fermenting food industry waste mixes have resulted in significant amounts of ε-pinene (12, 186). Additionally, *C. fimbriata* has been found to produce fruity aroma compounds from coffee waste, with steam-treated coffee husk providing an ideal fermentation substrate when supplemented with 20% glucose (155). In citrus waste applications, citric pulp waste from juice processing, when combined with soya bran and sugarcane molasses, has been successfully used to cultivate *C. fimbriata* for aroma compound production (29, 69). Similarly, studies using cassava plant bagasse as a substrate for *K. marxianus* in SSF found that oxygen availability significantly influenced total volatile compound production, particularly in packed bed reactors (186).

SSF has been favored over SmF for aroma compound production due to its ability to provide an optimal microbial growth environment and higher volatile yield. For example, *Trichoderma* sp. produced 6-pentyl-α-pyrone (6-PP), a compound associated with coconut aromas, more effectively under SSF conditions, with SCB as a dedicated support (156). The interactions between microbial metabolism and substrate composition determine the sensory attributes of the final product. Specific yeast strains, such as *Brettanomyces bruxellensis*, have been shown to extract phenylethyl alcohol (rose fragrance) from carrot, orange, and apple pomace, yielding 2.68 g/kg wet carrot pomace weight (80). Studies on orange pomace fermentation have identified floral-scented chemicals such as citronellyl formate, nerolidol, and cis-geraniol, while limonene, citral, and valencene contribute to citrus notes (5).

The physicochemical properties of vegetable food matrices influence flavor release and retention. Lipids found in fruit and vegetable peels contain hydrophobic flavor molecules, while proteins interact with volatiles via hydrophobic and electrostatic forces (157, 158). Processing methods such as fermentation, heating, and freezing further impact flavor dynamics (159, 160). The Maillard reaction, which occurs during thermal treatment, results in the formation of furans, pyrazines, and thiols, all of which contribute to complex aromas. Additionally, enzymatic activities release fruity, floral, and spicy volatile compounds (187). The selection of microbial strains remains a key factor in flavor production. Studies have found that *K. marxianus*-fermented vegetable pomaces contain higher concentrations of isovaleric acid, isoamyl acetate, and phenyl ethyl acetate, while *D. hansenii*-fermented pomaces exhibit greater amounts of methyl-2-methylpentanoate, demonstrating strain-specific flavor variations (161).

## 7 Conclusion

SSF of vegetable waste represents a powerful approach to flavor production, offering a sustainable alternative to conventional methods. By harnessing the metabolic activity of microorganisms, SSF enables the conversion of agro-industrial by-products into valuable aroma compounds, addressing both food industry demands and environmental concerns. The ability of fungi, yeasts, and bacteria to generate diverse flavor molecules from nutrient-rich waste underscores the potential of this process in reducing food loss while meeting the growing demand for natural ingredients. The success of SSF depends on multiple factors, including substrate composition, microbial selection, and process optimization. Careful control of fermentation parameters enhances microbial performance, leading to higher yields and improved sensory profiles. While SSF provides advantages such as lower water usage, enhanced product complexity, and sustainability, challenges remain in scalability, process consistency, and efficient compound recovery. Advances in microbial engineering, adaptive fermentation techniques, and bioseparation technologies will be essential in overcoming these hurdles and unlocking the full potential of SSF for industrial applications.

Future research should focus on optimizing microbial metabolic pathways, improving bioprocessing strategies for diverse feedstocks, and integrating SSF with innovative extraction techniques. A deeper understanding of the interactions between microorganisms and substrates will allow for greater precision in flavor development, ensuring reproducibility and economic feasibility. Ultimately, SSF stands at the intersection of biotechnology and sustainability, offering a practical and innovative solution for flavor generation. By transforming agricultural waste into high-value compounds, this process not only contributes to a more circular food system but also aligns with consumer preferences for natural and environmentally responsible products. With continued advancements, SSF has the potential to reshape the future of flavor production, bridging the gap between efficiency, sustainability, and sensory excellence.

## References

[1] Voilley A Simatos D. Flavor chemistry and technology. Food Chem. (2005) 98:436–42. doi: 10.1016/j.foodchem.2005.05.042

[2] Sharma R Ghoshal G. Emerging trends in food packaging. Nutr Food Sci. (2018) 50:294–305. doi: 10.1108/NFS-02-2018-0051

[3] Martínez-Ávila G Rodríguez-Jiménez G. Bio-production of natural flavors from agricultural waste. Bioeng Biotechnol. (2013) 3:122–30. doi: 10.1016/j.foodchem.2013.11.130

[4] Melini V Melini F Luziatelli F Ruzzi M. Functional ingredients from agri-food waste: effect of inclusion thereof on phenolic compound content and bioaccessibility in bakery products. Antioxidants. (2020) 9:1216. doi: 10.3390/antiox9121216, PMID: 33276525 PMC7761272

[5] Hadj Saadoun J Ghemmaz M. Transforming agricultural by-products into value-added flavor compounds. J Food Sci Technol. (2021) 58:12–23. doi: 10.1007/s13197-020-04554-6

[6] Aschemann-Witzel J Zielke S. Can't buy me green? A review of consumer perceptions of and behavior toward the price of organic food. J Food Prod Mark. (2017) 23:103–20. doi: 10.1111/joca.12092

[7] Vasilaki C Achim S Butinar B. Insights into flavor enhancers: definition, mechanism of action, and market trends. Crit Rev Food Sci Nutr. (2021) 62:1–12. doi: 10.1080/10408398.2021.1939264 34142890

[8] Inoue Y Kato S Saikusa M Suzuki C Otsubo Y Tanaka Y. Analysis of the cooked aroma and odorants that contribute to umami aftertaste of soy miso (Japanese soybean paste). Food Chem. (2016) 213:521–8. doi: 10.1016/j.foodchem.2016.06.106, PMID: 27451212

[9] Paulino BN Sales A Felipe LD Pastore GM Molina G Bicas JL. Biotechnological production of non-volatile flavor compounds. Curr Opin Food Sci. (2021) 41:26–35. doi: 10.1016/j.cofs.2021.02.003

[10] Bel-Rhlid R Günter Berger R Blank I. Bio-mediated generation of food flavors – towards sustainable flavor production inspired by nature. Trends Food Sci Technol. (2018) 78:134–43. doi: 10.1016/j.tifs.2018.06.004

[11] Chilakamarry CR Mimi Sakinah AM Zularisam A Sirohi R Ahamad Khilji I Ahmad NA. Advances in solid-state fermentation for bioconversion of agricultural wastes to value-added products: opportunities and challenges. Bioresour Technol. (2021) 343:126065. doi: 10.1016/j.biortech.2021.126065, PMID: 34624472

[12] Aggelopoulos T Katsieris K Bekatorou A Pandey A Banat IM Koutinas AA. Solid-state fermentation of food waste mixtures for single-cell protein, aroma volatiles, and fat production. Food Chem. (2014) 145:710–6. doi: 10.1016/j.foodchem.2013.07.105, PMID: 24128535

[13] Yazid NA Barrena R Sánchez A. Coconut husks as substrates for solid-state fermentation. J Clean Prod. (2016) 112:2303–9. doi: 10.1016/j.jclepro.2016.09.163

[14] Lucas S Fernández P Angosto JM. Food additives: function, safety, and regulatory aspects. Crit Rev Food Sci Nutr. (2021) 62:85–106. doi: 10.1080/10408398.2021.1929823

[15] Wu Y Xia M Zhao N Tu L Xue D Zhang X. Metabolic profile of main organic acids and its regulatory mechanism in solid-state fermentation of Chinese cereal vinegar. Food Res Int. (2021) 145:110400. doi: 10.1016/j.foodres.2021.110400, PMID: 34112403

[16] Chen QC Wang J. Simultaneous determination of artificial sweeteners, preservatives, caffeine, theobromine, and theophylline in food and pharmaceutical preparations by ion chromatography. J Chromatogr A. (2001) 937:57–64. doi: 10.1016/s0021-9673(01)01306-1, PMID: 11765085

[17] Schiffman S. Sensory enhancement of foods for the elderly with monosodium glutamate and flavors. Food Rev Int. (1998) 14:321–33. doi: 10.1080/87559129809541164

[18] Spence C Ngo MK. Assessing the shape symbolism of the taste, flavor, and texture of foods and beverages. Flavor. (2012) 1:12. doi: 10.1186/2044-7248-1-12

[19] Schiffman S. Intensification of sensory properties of foods for the elderly. J Nutr. (2000) 130:927S–30S. doi: 10.1093/jn/130.4.927S, PMID: 10736354

[20] Yamaguchi S. Basic properties of umami and its effects on food flavor. J Food Sci. (2002) 54:1074–7. doi: 10.53879/id.54.02.10705

[21] Nguyen T Lee J Lee KG. Regional and cultural influences on flavor preferences. Int J Food Sci. (2021) 56:200–12. doi: 10.47604/ijf.2123

[22] Haro-González JN Castillo-Herrera GA Martínez-Velázquez M Espinosa-Andrews H. Clove essential oil (*Syzygium aromaticum* L. Myrtaceae): extraction, chemical composition, food applications, and essential bioactivity for human health. Molecules. (2021) 26:6387. doi: 10.3390/molecules26216387, PMID: 34770801 PMC8588428

[23] Jayathilakan K Sultana K Radhakrishna K Bawa AS. Utilization of byproducts and waste materials from meat, poultry and fish processing industries: a review. J Food Sci Technol. (2012) 49:278–93. doi: 10.1007/s13197-011-0290-7, PMID: 23729848 PMC3614052

[24] Nelson HN Lange K Hunter S O'Bert A Wilson T. Characterization of vanilla taste preference. FASEB J. (2015) 29:LB251. doi: 10.1096/fasebj.29.1_supplement.lb251

[25] Li YX Erhunmwunsee F Liu M Yang K Zheng W Tian J. Antimicrobial mechanisms of spice essential oils and application in food industry. Food Chem. (2022) 382:132312. doi: 10.1016/j.foodchem.2022.132312, PMID: 35158267

[26] Jiang TA. Health benefits of culinary herbs and spices. J AOAC Int. (2019) 102:395–411. doi: 10.5740/jaoacint.18-0418, PMID: 30651162

[27] Sowbhagya H Chitra V. Enzyme-assisted extraction of flavorings and colorants from plant materials. Crit Rev Food Sci Nutr. (2010) 50:146–61. doi: 10.1080/10408390802248775, PMID: 20112157

[28] Zhang QW Lin LG Ye WC. Techniques for extraction and isolation of natural products: a comprehensive review. Chin Med. (2018) 13:20. doi: 10.1186/s13020-018-0177-x, PMID: 29692864 PMC5905184

[29] Mastelić J Jerković I Blažević I Radonić A Krstulović L. Hydrodistillation-adsorption method for the isolation of water-soluble, non-soluble and high volatile compounds from plant materials. Talanta. (2008) 76:885–91. doi: 10.1016/j.talanta.2008.04.051, PMID: 18656674

[30] Chibuye B Singh IS Luke C Kakoma MK. A review of modern and conventional extraction techniques and their applications for extracting phytochemicals from plants. Sci Afr. (2023) 19:e01585. doi: 10.1016/j.sciaf.2023.e01585

[31] Ferhat MA Boukhatem MN Hazzit M Meklati BY Chemat F. Cold pressing hydrodistillation and microwave dry distillation of citrus essential oil from Algeria: a comparative study. Electron J Biol. S1, 30–41. (2016)

[32] Lioe HN Selamat J Yasuda M. Soy sauce and its umami taste: a link from the past to current situation. J Food Sci. (2010) 75:R71–6. doi: 10.1111/j.1750-3841.2010.01529.x, PMID: 20492309

[33] Craig WJ Mangels AR Brothers CJ. Nutritional profiles of non-dairy plant-based cheese alternatives. Nutrients. (2022) 14:1247. doi: 10.3390/nu14061247, PMID: 35334904 PMC8952881

[34] Kumar VD Thyab Gddoa Al-Sahlany S Kareem Niamah A Thakur M Shah N Singh S. Recent trends in microbial flavor compounds: a review on chemistry, synthesis mechanism and their application in food. Saudi J Biol Sci. (2021) 29:1565–76. doi: 10.1016/j.sjbs.2021.11.010, PMID: 35280596 PMC8913424

[35] McGorrin R.J. Character impact compounds: flavors and off-flavors in foods. Taylor & Francis eBooks (2001).

[36] Chen L Li K Chen H Li Z. Reviewing the source, physiological characteristics, and aroma production mechanisms of aroma-producing yeasts. Food Secur. (2023) 12:3501. doi: 10.3390/foods12183501, PMID: 37761210 PMC10529235

[37] Gitaari MA Mwangi P Gitu LM. Chemical synthesis of vanillin from guaiacol and glyoxylic acid. Chem Sci Int J. (2019) 27:1–12. doi: 10.9734/csji/2019/v27i130104

[38] Furuya T Miura M Kino K. Efficient synthesis of vanillin using microbial fermentation of ferulic acid. Chembiochem. (2014) 15:795–803. doi: 10.1002/cbic.201402215 25164030

[39] Dong A Yu Z Pan T Rong L Lai M Cheng B. Synthesis and pyrolysis of ethyl maltol ester. Flavour Fragr J. (2023) 38:416–25. doi: 10.1002/ffj.3756

[40] Baines D Brown M. Flavor enhancers: characteristics and uses In: Encyclopedia of food and health. Cambridge, MA, USA: Elsevier (2016). 369–74.

[41] Yang Y Chen Q Shen C Zhang S Zhilin G Hu R. Evaluation of monosodium glutamate, disodium inosinate and guanylate umami taste by an electronic tongue. J Food Eng. (2013) 116:627–32. doi: 10.1016/j.jfoodeng.2012.12.042

[42] Olsman H. Hydrolyzed and autolyzed vegetable proteins as functional food ingredients. J Am Oil Chem Soc. (1979) 56:375–6. doi: 10.1007/bf02671502

[43] Vijayalakshmi S Disalva X Srivastava C Arun A. Vanilla-natural vs artificial: a review. Res J Pharm Technol. (2019) 12:01–05. doi: 10.5958/0974-360X.2019.00520.1

[44] Goodman MJ. The “natural” vs. “natural flavors” conflict in food labeling: a regulatory viewpoint. Food Drug Law J. (2017) 72:78–102. 29140655

[45] Murley T Chambers E 4th. The influence of colorants, flavorants and product identity on perceptions of naturalness. Food Secur. (2019) 8:317. doi: 10.3390/foods8080317, PMID: 31382670 PMC6722695

[46] Valenzuela JL. Advances in postharvest preservation and quality of fruits and vegetables. Food Secur. (2023) 12:1830. doi: 10.3390/foods12091830, PMID: 37174367 PMC10178206

[47] Tural S Aytaç B Özçelik B. Safety assessment of monosodium glutamate in food and its effects on human health. Rev Environ Health. (2021) 36:355–62. doi: 10.1515/reveh-2021-0158

[48] Nielsen EE Egebjerg MM Pedersen GA Sharma AK Olesen P Hansen M. Risk assessment of formaldehyde present in food and drinking water. Toxicol Lett. (2018) 295:12–48. doi: 10.1016/j.toxlet.2018.06.819

[49] Andreozzi L Giannetti A Cipriani F Caffarelli C Mastrorilli C Ricci G. Hypersensitivity reactions to food and drug additives: problem or myth? Acta Bio Med Atenei Parmensis. (2019) 90:80–90. doi: 10.23750/abm.v90i3-S.8168, PMID: 30830065 PMC6502174

[50] Ranadive AS. Vanillin and related flavor compounds in vanilla extracts made from beans of various global origins. J Agric Food Chem. (1992) 40:1922–4. doi: 10.1021/jf00022a039

[51] Rao S Ravishankar GA. Vanilla flavor: production by conventional and biotechnological routes. J Sci Food Agric. (2000) 80:289–304. doi: 10.1002/1097-0010(200002)80:3<289::aid-jsfa543>3.0.co;2-2

[52] Walton K Walker R van de Sandt JJ Castell JV Knapp AG Kozianowski G. The application of in vitro data in the derivation of the acceptable daily intake of food additives. Food Chem Toxicol. (1999) 37:1–42. doi: 10.1016/S0278-6915(99)00073-4 10654594

[53] Rouquié D Friry-Santini C Schorsch F Tinwell H Bars R. Standard and molecular NOAELs for rat testicular toxicity induced by flutamide. Toxicol Sci. (2009) 109:59–65. doi: 10.1093/toxsci/kfp056, PMID: 19299419

[54] Mortensen A Aguilar F Crebelli R Di Domenico A Dusemund B Franz R. Re-evaluation of glutamic acid–glutamates (E 620–625) as food additives. EFSA J. (2017) 15:4910. doi: 10.2903/j.efsa.2017.4910 PMC7009848 32625571

[55] Gaylor DW Bolger P Schwetz BA. U.S. food and drug administration perspective of the inclusion of effects of low-level exposures in safety and risk assessment. Environ Health Perspect. (1998) 106:391–4. 9539036 10.1289/ehp.98106s1391 PMC1533277

[56] Gaylor DW Kodell RL. Inclusion of effects of low-level exposures in safety assessments for threshold-mediated toxicities. Environ Health Perspect. (1999) 106:391–4. doi: 10.1289/ehp.98106s1391 PMC1533277 9539036

[57] Younes M Aggett P Aguilar F Crebelli R Di Domenico A Dusemund B. Re-evaluation of silicon dioxide (E 551) as a food additive. EFSA J. (2018) 16:5088. doi: 10.2903/j.efsa.2018.5088 PMC7009582 32625658

[58] Trasande L Shaffer R Sathyanarayana S. Food additives and child health. Pediatrics. (2018) 142:e20181410. doi: 10.1542/peds.2018-1410, PMID: 30037972 PMC6298598

[59] Baker MT Lu P Parrella JA Leggette HR. Investigating the effect of consumers’ knowledge on their acceptance of functional foods: a systematic review and meta-analysis. Food Secur. (2022) 11:1135. doi: 10.3390/foods11081135, PMID: 35454722 PMC9028956

[60] Güngörmüş C. Kılıç A. The safety assessment of food additives by reproductive and developmental toxicity studies. IntechOpen. (2012).

[61] Manan M Webb C. Design aspects of solid-state fermentation as applied to microbial bioprocessing. J Appl Biotechnol Bioeng. (2017) 4:1–22. doi: 10.15406/jabb.2017.04.00094

[62] Jin G Boeschoten S Hageman J Zhu Y Wijffels R Rinzema A. Identifying variables influencing traditional food solid-state fermentation by statistical modeling. Food Secur. (2024) 13:1317. doi: 10.3390/foods13091317, PMID: 38731688 PMC11083392

[63] Liu D Zhu Y Beeftink R Ooijkaas L Rinzema A Chen J. Chinese vinegar and its solid-state fermentation process. Food Rev Int. (2004) 20:407–24. doi: 10.1081/FRI-200033460

[64] Jin G Zhao Y Xin S Li T Xu Y. Solid-state fermentation engineering of traditional Chinese fermented food. Food Secur. (2024) 13:3003. doi: 10.3390/foods13183003, PMID: 39335930 PMC11430836

[65] Song Z Du H Zhang Y Xu Y. Unraveling core functional microbiota in traditional solid-state fermentation by high-throughput amplicons and metatranscriptomics sequencing. Front Microbiol. (2017) 8:1294. doi: 10.3389/fmicb.2017.01294, PMID: 28769888 PMC5509801

[66] Krishna C. Solid-state fermentation systems-an overview. Crit Rev Biotechnol. (2005) 25:1–30. doi: 10.1080/07388550590925383, PMID: 15999850

[67] Anal A Perpetuini G Petchkongkaew A Tan R Avallone S Tofalo R. Food safety risks in traditional fermented food from South-East Asia. Food Control. (2020) 109:106922. doi: 10.1016/j.foodcont.2019.106922

[68] Skowron K Budzyńska A Grudlewska-Buda K Wiktorczyk-Kapischke N Andrzejewska M Wałecka-Zacharska E. Two faces of fermented foods—the benefits and threats of its consumption. Front Microbiol. (2022) 13:845166. doi: 10.3389/fmicb.2022.845166, PMID: 35330774 PMC8940296

[69] Sharma A Sharma P Singh J Singh S Nain L. Prospecting the potential of agroresidues as substrate for microbial flavor production. Front Sustain Food Syst. (2020) 4:18. doi: 10.3389/fsufs.2020.00018

[70] Mannaa M Han G Seo YS Park I. Evolution of food fermentation processes and the use of multi-omics in deciphering the roles of the microbiota. Food Secur. (2021) 10:2861. doi: 10.3390/foods10112861, PMID: 34829140 PMC8618017

[71] Desnoyers M Giger-Reverdin S Bertin G Duvaux-Ponter C Sauvant D. Meta-analysis of the influence of *Saccharomyces cerevisiae* supplementation on ruminal parameters and milk production of ruminants. J Dairy Sci. (2009) 92:1620–32. doi: 10.3168/jds.2008-1414, PMID: 19307644

[72] Priefert H Rabenhorst J Steinbüchel A. Biotechnological production of vanillin. Appl Microbiol Biotechnol. (2001) 56:296–314. doi: 10.1007/s002530100687, PMID: 11548997

[73] Mostafa S Wang Y Zeng W Jin B. Floral scents and fruit aromas: functions, compositions, biosynthesis, and regulation. Front Plant Sci. (2022) 13:860157. doi: 10.3389/fpls.2022.860157, PMID: 35360336 PMC8961363

[74] Gupta C. A biotechnological approach to microbial-based perfumes and flavors. J Microbiol Exp. (2015) 2:11–8. doi: 10.15406/jmen.2015.02.00034

[75] Li T Liu X Xiang H Zhu H Lu X Feng B. Two-phase fermentation systems for microbial production of plant-derived terpenes. Molecules. (2024) 29:1127. doi: 10.3390/molecules29051127, PMID: 38474639 PMC10934027

[76] Braga A Guerreiro C Belo I. Generation of flavors and fragrances through biotransformation and de novo synthesis. Food Bioprocess Technol. (2018) 11:2217–28. doi: 10.1007/s11947-018-2180-8

[77] Ninkuu V Zhang L Yan J Fu Z Yang T Zeng H. Biochemistry of terpenes and recent advances in plant protection. Int J Mol Sci. (2021) 22:5710. doi: 10.3390/ijms22115710, PMID: 34071919 PMC8199371

[78] Zhang H Zhang L Yu X Xu Y. The biosynthesis mechanism involving 2,3-pentanedione and aminoacetone describes the production of 2-ethyl-3,5-dimethylpyrazine and 2-ethyl-3,6-dimethylpyrazine by *Bacillus subtilis*. J Agric Food Chem. (2020) 68:3558–67. doi: 10.1021/acs.jafc.9b07809, PMID: 32065523

[79] Mitchell DA Berovič M Krieger N. Solid-state fermentation bioreactor fundamentals: introduction and overview In: Mitchell DA Berovič M Krieger N, editors. Solid-state fermentation bioreactors. Berlin, Heidelberg, Germany: Springer (2006). 1.

[80] Lindsay M Granucci N Greenwood D Villas-Bôas S. Identification of new natural sources of flavor and aroma metabolites from solid-state fermentation of agro-industrial by-products. Meta. (2022) 12:157. doi: 10.3390/metabo12020157, PMID: 35208231 PMC8877680

[81] López-Gómez J Venus J. Potential role of sequential solid-state and submerged-liquid fermentations in a circular bioeconomy. Fermentation. (2021) 7:76. doi: 10.3390/FERMENTATION7020076

[82] Abu Yazid N Barrena R Komilis D Sánchez A. Solid-state fermentation as a novel paradigm for organic waste valorization: a review. Sustain For. (2017) 9:224. doi: 10.3390/su9020224

[83] De Oliveira B Coradi G De Oliva-Neto P Nascimento V. Biocatalytic benefits of immobilized fusarium sp. (GFC) lipase from solid-state fermentation on free lipase from submerged *fermentation*. Ind Crop Prod. (2020) 147:112235. doi: 10.1016/j.indcrop.2020.112235

[84] Hölker U Höfer M Lenz J. Biotechnological advantages of laboratory-scale solid-state fermentation with fungi. Appl Microbiol Biotechnol. (2004) 64:175–86. doi: 10.1007/s00253-003-1504-3, PMID: 14963614

[85] Viniegra González G FavelaTorres E Aguilar CN RomeroGómez SD DíazGodínez G Augur C. Advantages of fungal enzyme production in solid state over liquid fermentation systems. Biochem Eng J. (2003) 13:157–67. doi: 10.1016/S1369-703X(02)00128-6

[86] Hölker U Lenz J. Solid-state fermentation--are there any biotechnological advantages? Curr Opin Microbiol. (2005) 8:301–6. doi: 10.1016/J.MIB.2005.04.006, PMID: 15939353

[87] Verma S Daverey A Sharma A. Slow sand filtration for water and wastewater treatment – a review. Environ Technol Rev. (2017) 6:47–58. doi: 10.1080/21622515.2016.1278278

[88] Robinson T Singh D Nigam P. Solid-state fermentation: a promising microbial technology for secondary metabolite production. Appl Microbiol Biotechnol. (2001) 55:284–9. doi: 10.1007/s002530000565, PMID: 11341307

[89] Singhania RR Patel AK Soccol CR Pandey A. Recent advances in solid-state fermentation. Biochem Eng J. (2009) 44:13–8. doi: 10.1016/j.bej.2008.10.019

[90] Martins S Mussatto SI Martínez-Ávila G Montañez-Saenz J Aguilar CN Teixeira JA. Bioactive phenolic compounds: production and extraction by solid-state fermentation. A review. Biotechnol Adv. (2011) 29:365–73. doi: 10.1016/j.biotechadv.2011.01.008, PMID: 21291993

[91] Orzua MC Mussatto SI Contreras-Esquivel JC Rodriguez R de La Garza H Teixeira JA. Exploitation of agro industrial wastes as immobilization carrier for solid-state fermentation. Ind Crop Prod. (2009) 30:24–7. doi: 10.1016/j.indcrop.2009.02.001

[92] Sadh PK Duhan S Duhan JS. Agro-industrial wastes and their utilization using solid state fermentation: a review. Bioresour Bioprocess. (2018) 5:1–15. doi: 10.1186/s40643-017-0187-z

[93] Rabinovich GY Fomicheva NV. Development of carbon-transforming microorganisms in express fermentation processes with the use of food wastes. Russ Agricult Sci. (2007) 33:166–8. doi: 10.3103/S1068367407030093

[94] Thapak P Tripathi N Upadhyay R. Isolation and partial identification of bacteria and fungi from fermenting vegetable garbage of kitchen. Pharm Biosci J. (2019). 7, 05–09. doi: 10.20510/ukjpb/7/i5/1567589404

[95] Zheng Y Mou J Niu J Yang S Chen L Xia M. Succession sequence of lactic acid bacteria driven by environmental factors and substrates throughout the brewing process of Shanxi aged vinegar. Appl Microbiol Biotechnol. (2018) 102:2645–58. doi: 10.1007/s00253-017-8733-3, PMID: 29430584

[96] Bolaji IO Dionisi D. Acidogenic fermentation of vegetable and salad waste for chemicals production: effect of pH buffer and retention time. J Environ Chem Eng. (2017) 5:5933–43. doi: 10.1016/j.jece.2017.11.001

[97] Cheah YK Dosta J Mata-Álvarez J. Enhancement of volatile fatty acids production from food waste by mature compost addition. Molecules. (2019) 24:2986. doi: 10.3390/molecules24162986, PMID: 31426488 PMC6721731

[98] Lu Y Zhang Q Wang X Zhou X Zhu J. Effect of pH on volatile fatty acid production from anaerobic digestion of potato peel waste. Bioresour Technol. (2020) 316:123851. doi: 10.1016/j.biortech.2020.123851, PMID: 32738559

[99] Chen H Wu J Huang R Zhang W He W Deng Z. Effects of temperature and total solid content on biohydrogen production from dark fermentation of rice straw: performance and microbial community characteristics. Chemosphere. (2021) 286:131655. doi: 10.1016/j.chemosphere.2021.131655 34315083

[100] Pandey A. Solid-state fermentation. Biochem Eng J. (2003) 13:81–4. doi: 10.1016/S1369-703X(02)00121-3, PMID: 10908866

[101] Abraham J Gea T Sánchez A. Substitution of chemical dehairing by proteases from solid-state fermentation of hair wastes. J Clean Prod. (2014) 74:191–8. doi: 10.1016/j.jclepro.2014.03.035

[102] Jiang Y Xin F Lu J Dong W Zhang W Zhang M. State of the art review of biofuels production from lignocellulose by thermophilic bacteria. Bioresour Technol. (2017) 245:1498–506. doi: 10.1016/j.biortech.2017.05.142, PMID: 28634129

[103] Cai S Wang O Wu W Zhu S Zhou F Ji B. Comparative study of the effects of solid-state fermentation with three filamentous fungi on the total phenolics content (TPC), flavonoids, and antioxidant activities of subfractions from oats (*Avena sativa* L.). J Agric Food Chem. (2012) 60:507–13. doi: 10.1021/jf204163a, PMID: 22136169

[104] Crafack M Keul H Eskildsen CE Petersen MA Saerens S Blennow A. Impact of starter cultures and fermentation techniques on the volatile aroma and sensory profile of chocolate. Food Res Int. (2014) 63:306–16. doi: 10.1016/j.foodres.2014.04.032

[105] Juanssilfero AB Kahar P Amza R Miyamoto N Otsuka H Matsumoto H. Effect of inoculum size on single-cell oil production from glucose and xylose using oleaginous yeast Lipomyces starkeyi. J Biosci Bioeng. (2018) 125:695–702. doi: 10.1016/j.jbiosc.2017.12.020 29373308

[106] Norazlina I Ku Halim KH Abd Manaf SF Abu Bakar MA. Effect of carbon and nitrogen ratio, mineral solution & inoculum size in the production of xylanase using oil palm leaf. Adv Mater Res. (2015) 1113:273–8. doi: 10.4028/www.scientific.net/amr.1113.273

[107] Carrau F Medina K Fariña L Boido E Dellacassa E. Effect of *Saccharomyces cerevisiae* inoculum size on wine fermentation aroma compounds and its relation with assimilable nitrogen content. Int J Food Microbiol. (2010) 143:81–5. doi: 10.1016/j.ijfoodmicro.2010.07.024, PMID: 20692063

[108] Arora S Rani R Ghosh S. Bioreactors in solid-state fermentation technology: design, applications, and engineering aspects. J Biotechnol. (2018) 269:16–34. doi: 10.1016/j.jbiotec.2018.01.010, PMID: 29408199

[109] Perez C Casciatori F Thoméo J. Strategies for scaling-up packed-bed bioreactors for solid-state fermentation: the case of cellulolytic enzymes production by a thermophilic fungus. Chem Eng J. (2019) 35:167–78. doi: 10.1016/J.CEJ.2018.12.169

[110] Abdeshahian P Ascencio JJ Philippini RR Antunes FAF de Carvalho AS Abdeshahian M. Valorization of lignocellulosic biomass and agri-food processing wastes for production of glucan polymer. Waste Biomass Valor. (2021) 12:2915–31. doi: 10.1007/s12649-020-01267-z

[111] Rai S Dutta PK Mehrotra GK. Natural antioxidant and antimicrobial agents from agrowastes: an emergent need to food packaging. Waste Biomass Valor. (2020) 11:1905–16. doi: 10.1007/s12649-018-0498-0

[112] Augustin MA Riley M Stockmann R Bennett L Kahl A Lockett T. Role of food processing in food and nutrition security. Trends Food Sci Technol. (2016) 56:115–25. doi: 10.1016/J.TIFS.2016.08.005

[113] Bharathiraja S Suriya J Krishnan M Manivasagan P Kim SK. Production of enzymes from agricultural wastes and their potential industrial applications. Adv Food Nutr Res. (2017) 80:125–48. doi: 10.1016/bs.afnr.2016.11.003, PMID: 28215322

[114] Valdez-Arjona L Ramírez-Mella M. Pumpkin waste as livestock feed: impact on nutrition and animal health and on quality of meat, milk, and egg. Animals (Basel). (2019) 9:769. doi: 10.3390/ani9100769, PMID: 31597395 PMC6826842

[115] Zepf F Jin B. Bioconversion of grape marc into protein rich animal feed by microbial fungi. Chem Eng Process Tech. (2013) 1:1011–8.

[116] Rodríguez Couto S. Exploitation of biological wastes for the production of value-added products under solid-state fermentation conditions. Biotechnol J. (2008) 3:859–70. doi: 10.1002/biot.200800031, PMID: 18543242

[117] Martínez-Ávila O Muñoz-Torrero P Sánchez A Font X Barrena R. Valorization of agro-industrial wastes by producing 2-phenylethanol via solid-state fermentation: influence of substrate selection on the process. Waste Manag. (2021) 121:403–11. doi: 10.1016/j.wasman.2020.12.036, PMID: 33445113

[118] Chen H He Q. Value-added bioconversion of biomass by solid-state fermentation. J Chem Technol Biotechnol. (2012) 87:1619–25. doi: 10.1002/jctb.3901

[119] Pagliano G Ventorino V Panico A Pepe O. Integrated systems for biopolymers and bioenergy production from organic waste and by-products: a review of microbial processes. Biotechnol Biofuels. (2017) 10:113. doi: 10.1186/s13068-017-0802-4, PMID: 28469708 PMC5414342

[120] Mantzouridou FT Paraskevopoulou A Lalou S. Yeast flavor production by solid-state fermentation of orange peel waste. Biochem Eng J. (2015) 101:1–8. doi: 10.1016/j.bej.2015.04.013

[121] Wang Z Lu Z Shi J Xu Z. Exploring flavour-producing core microbiota in multispecies solid-state fermentation of traditional Chinese vinegar. Sci Rep. (2016) 6:26818. doi: 10.1038/srep26818, PMID: 27241188 PMC4886211

[122] Harding MW Marques LL Howard RJ Olson ME. Can filamentous fungi form biofilms? Trends Microbiol. (2009) 17:475–80. doi: 10.1016/j.tim.2009.08.007, PMID: 19833519

[123] Zhang K Zhang T Guo R Ye Q Zhao H Huang X. The regulation of key flavor of traditional fermented food by microbial metabolism: a review. Food Chem X. (2023) 19:100871. doi: 10.1016/j.fochx.2023.100871, PMID: 37780239 PMC10534219

[124] Bhunia B Basak B Dey A. A review on production of serine alkaline protease by Bacillus spp. J Biochem Technol. (2012) 3:448–57.

[125] Lee DE Lee S Jang ES Shin HW Moon BS Lee CH. Metabolomic profiles of aspergillus oryzae and *Bacillus amyloliquefaciens* during rice koji fermentation. Molecules. (2016) 21:773. doi: 10.3390/molecules21060773, PMID: 27314317 PMC6273993

[126] Steinkraus K. Fermentations in world food processing. Compr Rev Food Sci Food Saf. (2002) 1:23–32. doi: 10.1111/J.1541-4337.2002.TB00004.X, PMID: 33451246

[127] Ramachandran P Alawaed A Singh A. Titanium-mediated reduction of carboxamides to amines with borane–ammonia. Molecules. (2023) 28:4575. doi: 10.3390/molecules28124575, PMID: 37375131 PMC10301125

[128] Dai Z Wu Z Zhu W Wu G. Amino acids in microbial metabolism and function. Adv Exp Med Biol. (2022) 1354:127–43. doi: 10.1007/978-3-030-85686-1_7, PMID: 34807440

[129] Li T Kootstra A Fotheringham I. Nonproteinogenic α-amino acid preparation using equilibrium shifted transamination. Org Process Res Dev. (2002) 6:533–8. doi: 10.1021/OP025518X

[130] Cho BK Seo JH Kim J Lee CS Kim BG. Asymmetric synthesis of unnatural l-amino acids using thermophilic aromatic l-amino acid transaminase. Biotechnol Bioprocess Eng. (2006) 11:299–305. doi: 10.1007/BF03026244

[131] Rijnen L Bonneau S Yvon M. Genetic characterization of the major lactococcal aromatic aminotransferase and its involvement in conversion of amino acids to aroma compounds. Appl Environ Microbiol. (1999) 65:4873–80. doi: 10.1128/AEM.65.11.4873-4880.1999, PMID: 10543798 PMC91656

[132] Lee J Heo S Choi J Kim M Pyo E Lee M. Selection of *Lactococcus lactis* HY7803 for glutamic acid production based on comparative genomic analysis. J Microbiol Biotechnol. (2020) 31:298–303. doi: 10.4014/jmb.2011.11022 PMC9705870 33397831

[133] Shahidi F Hossain A. Role of lipids in food flavor generation. Molecules (Basel, Switzerland). (2022) 27:5014. doi: 10.3390/molecules27155014, PMID: 35956962 PMC9370143

[134] Khadka Y. Carbohydrates—a brief deliberation with bio-aspect. Cognition. (2022) 4:125–38. doi: 10.3126/cognition.v4i1.46484

[135] Xia Y Yu J Liu H Feng C Shuang Q. Novel insight into physicochemical and flavor formation in koumiss based on microbial metabolic network. Food Res Int. (2021) 149:110659. doi: 10.1016/j.foodres.2021.110659, PMID: 34600661

[136] Zhang Y Liu M Cai B He K Wang M Chen B. De novo biosynthesis of α-aminoadipate via multi-strategy metabolic engineering in *Escherichia coli*. Microbiol Open. (2022) 11:e1301. doi: 10.1002/mbo3.1301, PMID: 36314756 PMC9437556

[137] Wang H Liao X Lin C Bai W Xiao G Huang X. Optimization of fermentation conditions, physicochemical profile and sensory quality analysis of seedless wampee wine. Appl Biol Chem. (2024) 67:81. doi: 10.1186/s13765-024-00938-y

[138] Correia-Lima L Da-Silva JR Fernandes GL Ribeiro-Filho N Madruga MS Lima MD. Applying beet peels as a brewing adjunct and its impact on flavor formation. Int J Gastron Food Sci. (2023) 32:100846. doi: 10.1016/j.ijgfs.2023.100846

[139] Wiegand S Voigt B Albrecht D Bongaerts J Evers S Hecker M. Fermentation stage-dependent adaptations of *Bacillus licheniformis* during enzyme production. Microb Cell Factories. (2013) 12:120. doi: 10.1186/1475-2859-12-120, PMID: 24313996 PMC3878961

[140] Venkataraman S Athilakshmi JK Rajendran DS Bharathi P Kumar VV. A comprehensive review of eclectic approaches to the biological synthesis of vanillin and their application towards the food sector. Food Sci Biotechnol. (2024) 33:1019–36. doi: 10.1007/s10068-023-01484-x, PMID: 38440686 PMC10908958

[141] Tupe RV Singh NK Odaneth AA. Biotransformation of maize bran-derived ferulic acid to vanillin using an adapted strain of *Amycolatopsis* sp. ATCC 39116. Biotechnol Prog. (2024) 40:e3417. doi: 10.1002/btpr.3417, PMID: 38415921

[142] Saeed S Ur Rehman Baig U Tayyab M Altaf I Irfan M Raza SQ. Valorization of banana peels waste into biovanillin and optimization of process parameters using submerged fermentation. Biocatal Agric Biotechnol. (2021) 36:102154. doi: 10.1016/j.bcab.2021.102154

[143] Boccia F Punzo G. A choice experiment on consumer perceptions of three generations of genetically modified foods. Appetite. (2021) 161:105158. doi: 10.1016/j.appet.2021.105158, PMID: 33561496

[144] Gallage N Møller B. Vanillin-bioconversion and bioengineering of the most popular plant flavor and its de novo biosynthesis in the vanilla orchid. Mol Plant. (2014) 8:40–57. doi: 10.1016/j.molp.2014.11.008 25578271

[145] Ayivi RD Ibrahim SA. Lactic acid bacteria: an essential probiotic and starter culture for the production of yoghurt. Int J Food Sci Technol. (2022) 57:7008–25. doi: 10.1111/ijfs.16076

[146] Hu Y Zhang L Wen R Chen Q Kong B. Role of lactic acid bacteria in flavor development in traditional Chinese fermented foods: a review. Crit Rev Food Sci Nutr. (2020) 62:2741–55. doi: 10.1080/10408398.2020.1858269, PMID: 33377402

[147] Chen C Zhao S Hao G Yu H Tian H Zhao G. Role of lactic acid bacteria on yogurt flavor: a review. Int J Food Prop. (2017) 20:S316–30. doi: 10.1080/10942912.2017.1295988

[148] Sun H Du J Yan X Chen X Zhao L. Dynamic changes in aromas and precursors of edible fungi juice: mixed lactic acid bacteria fermentation enhances flavor characteristics. J Sci Food Agric. (2024) 104:8541–52. doi: 10.1002/jsfa.13681, PMID: 39392670

[149] Ayivi RD Edwards A Carrington D Brock A Krastanov A Eddin AS. The cultivation, growth, and viability of lactic acid bacteria: a quality control perspective. J Vis Exp. (2022) 184:e63314. doi: 10.3791/63314 35781528

[150] Güneşer O Demirkol A Karagül Yüceer Y Özmen Toğay S İşleten Hoşoğlu M Elibol M. Bioflavour production from tomato and pepper pomaces by *Kluyveromyces marxianus* and *Debaryomyces hansenii*. Bioprocess Biosyst Eng. (2015) 38:1143–55. doi: 10.1007/s00449-015-1356-0, PMID: 25614449

[151] Spaggiari M Ricci AM Calani L Bresciani L Neviani E Dall’Asta C. Solid state lactic acid fermentation: a strategy to improve wheat bran functionality. LWT. (2020) 118:108668. doi: 10.1016/j.lwt.2019.108668

[152] Liu G Huang L Lian J. Alcohol acyltransferases for the biosynthesis of esters. Biotechnol Biofuels Bioprod. (2023) 16:93. doi: 10.1186/s13068-023-02343-x, PMID: 37264424 PMC10236719

[153] Siddiqui SA Erol Z Rugji J Taşçı F Kahraman HA Toppi V. An overview of fermentation in the food industry: looking back from a new perspective. Bioresour Bioprocess. (2023) 10:85. doi: 10.1186/s40643-023-00702-y, PMID: 38647968 PMC10991178

[154] Akacha N Gargouri M. Microbial and enzymatic technologies used for the production of natural aroma compounds: synthesis, recovery modeling, and bioprocesses. Food Bioprod Process. (2015) 94:675–706. doi: 10.1016/j.fbp.2014.09.011

[155] Soares M Christen P Pandey A Soccol C. Fruity flavour production by *Ceratocystis fimbriata* grown on coffee husk in solid-state fermentation. Process Biochem. (2000) 35:857–61. doi: 10.1016/S0032-9592(99)00144-2

[156] Ladeira N Peixoto V Penha M Barros E Leite S. Optimization of 6-pentyl-alpha-pyrone production by solid state fermentation using sugarcane bagasse as residue. Bioresources. (2010) 5:2297–2306. doi: 10.15376/BIORES.5.4.2297-2306

[157] Dias PG Sajiwanie J Rathnayaka R. Chemical composition, physicochemical, and technological properties of selected fruit peels as a potential food source. Int J Fruit Sci. (2020) 20:S240–51. doi: 10.1080/15538362.2020.1717402

[158] Pathak P Mandavgane S Kulkarni B. Fruit peel waste: characterization and its potential uses. Curr Sci. (2017) 113:444–54. doi: 10.18520/CS/V113/I03/444-454

[159] Henríquez CA Cordova A Almonacid SF Saavedra J. Kinetic modeling of phenolic compound degradation during drum-drying of apple peel by-products. J Food Eng. (2014) 143:146–53. doi: 10.1016/J.JFOODENG.2014.06.037

[160] Nurlatifah I. Agustine D. Puspasari E. Production and characterization of eco-enzyme from fruit peel waste. Proceedings of the 1st international conference on social, science, and technology, ICSST 2021, 25. (2022).

[161] Güneşer O Yuceer Y Hosoglu MI Toğay SO. Production of some higher alcohols and acetate esters from rice bran by yeasts metabolisms of *Kluyveromyces marxianus* and *Debaryomyces hansenii*. Bioresour Technol. (2021) 01–15. doi: 10.21203/RS.3.RS-198090/V1 PMC9433634 35488980

[162] Furuya T Miura M Kuroiwa M Kino K. High-yield production of vanillin from ferulic acid by a coenzyme-independent decarboxylase/oxygenase two-stage process. New Biotechnol. (2015) 32:335–9. doi: 10.1016/j.nbt.2015.03.002, PMID: 25765579

[163] Li J Di T Bai J. Distribution of volatile compounds in different fruit structures in four tomato cultivars. Molecules. (2019) 24:2594. doi: 10.3390/molecules24142594, PMID: 31319482 PMC6681445

[164] Marella ER Dahlin J Dam MI ter Horst J Christensen HB Sudarsan S. A single-host fermentation process for the production of flavor lactones from non-hydroxylated fatty acids. Metab Eng. (2020) 61:427–36. doi: 10.1016/j.ymben.2019.08.009, PMID: 31404648

[165] Martínez-Ávila O Sánchez A Font X Barrena R. 2-phenylethanol (rose aroma) production potential of an isolated *Pichia kudriavzevii* through solid-state fermentation. Process Biochem. (2020) 93:94–103. doi: 10.1016/j.procbio.2020.03.023

[166] Smit G Smit BA Engels WJM. Flavour formation by lactic acid bacteria and biochemical flavour profiling of cheese products. FEMS Microbiol Rev. (2005) 29:591–610. doi: 10.1016/j.fmrre.2005.04.002, PMID: 15935512

[167] Molina G Pessôa MG Bicas JL Fontanille P Larroche C Pastore GM. Optimization of limonene biotransformation for the production of bulk amounts of α-terpineol. Bioresour Technol. (2019) 294:122180. doi: 10.1016/j.biortech.2019.122180, PMID: 31606595

[168] Yang Y Zhang M Hua J Deng Y Jiang Y Li J. Quantitation of pyrazines in roasted green tea by infrared-assisted extraction coupled to headspace solid-phase microextraction in combination with GC-QqQ-MS/MS. Food Res Int. (2020) 134:109167. doi: 10.1016/j.foodres.2020.109167, PMID: 32517930

[169] Barciela P Perez-Vazquez A Prieto MA. Azo dyes in the food industry: features, classification, toxicity, alternatives, and regulation. Food Chem Toxicol. (2023) 178:113935. doi: 10.1016/j.fct.2023.113935, PMID: 37429408

[170] Krishna C Nokes S. Influence of inoculum size on phytase production and growth in solid-state fermentation by *Aspergillus niger*. Trans ASABE. (2001) 44:1031–6. doi: 10.13031/2013.6224

[171] Madrera RR Bedriñana RM Valles BS. Production and characterization of aroma compounds from apple pomace by solid-state fermentation with selected yeasts. LWT-Food Sci Technol. (2015) 64:1342–53. doi: 10.1016/j.foodchem.2010.11.129

[172] Bramorski A Soccol C Soccol C Christen P Revah S. Fruit aroma production by *Ceratocystis fimbriata* in solid cultures from agroindustrial wastes. Ind Microbiol. (1998) 29:208–12. doi: 10.1590/S0001-37141998000300012

[173] Quilter MG Hurley JC Lynch FJ Murphy MG. The production of isoamyl acetate from amyl alcohol by *Saccharomyces cerevisiae*. J Inst Brew. (2003) 109:34–40. doi: 10.1002/j.2050-0416.2003.tb00591.x

[174] Briand L. Salles C. (2016). Taste perception and integration. In Flavor: From food to behaviors, wellbeing and health. Woodhead Publishing. 101–119.

[175] Castro-Muñoz R. Correa-Delgado M. Córdova-Almeida R. Lara-Nava D. Chávez-Muñoz M. Velásquez-Chávez V. F.. (2022). Natural sweeteners: Sources, extraction and current uses in foods and food industries. Food Chemistry, 370, 130991. doi: 10.1016/j.foodchem.2021.130991 34509947

[176] Tisserand R. Young R. (2014). Essential oil safety: A guide for health care professionals (2nd ed.). Churchill Livingstone.

[177] Gao P. Jiang Q. Xu Y. Xia W. (2018). Biosynthesis of acetate esters by dominate strains, isolated from Chinese traditional fermented fish (Suan yu). Food Chemistry, 244, 44–49. doi: 10.1016/j.foodchem.2017.10.007 29120803

[178] Altwasser V. Pätz R. R. Lemke T. Paufler S. Maskow T. (2016). A simple method for the measurement of metabolic heat production rates during solid-state fermentations using β-carotene production with *Blakeslea trispora* as a model system. Engineering in Life Sciences, 17, 620–628. doi: 10.1002/elsc.201600208 PMC6999528 32624807

[179] Wang Y. Chen Y. Xin J. Chen X. Xu T. He J.. (2023). Metabolomic profiles of the liquid state fermentation in co-culture of *Eurotium amstelodami* and Bacillus licheniformis. Frontiers in Microbiology, 14, 1080743. doi: 10.3389/fmicb.2023.1080743 36778878 PMC9909110

[180] Zhong A. Chen W. Duan Y. Li K. Tang X. Tian X.. (2021). The potential correlation between microbial communities and flavors in traditional fermented sour meat. LWT - Food Science and Technology, 149, 111873. doi: 10.1016/j.lwt.2021.111873

[181] Lei H. Liu X. Zhao W. Lin S. Lin J. Li J. (2024). Sea bass fish head broth treated by thermo-ultrasonication: Improving the nutritional properties and emulsion stability. Foods, 13, 2498. doi: 10.3390/foods13162498 39200425 PMC11354003

[182] Melini F. Melini V. (2024). Role of microbial fermentation in the bio-production of food aroma compounds from vegetable waste. Fermentation, 10, 132. doi: 10.3390/fermentation10030132

[183] Ma X.-k. Daugulis A. J. (2014). Effect of bioconversion conditions on vanillin production by *Amycolatopsis* sp. ATCC 39116 through an analysis of competing by-product formation. Bioprocess and Biosystems Engineering, 37, 891–899. doi: 10.1007/s00449-013-1060-x 24078147

[184] Li M. Li B. Zhang W. (2018). Rapid and non-invasive detection and imaging of the hydrocolloid-injected prawns with low-field NMR and MRI. Food Chemistry, 242, 16–21. doi: 10.1016/j.foodchem.2017.08.086 29037672

[185] Mehmood T. Ahmed S. Waseem R. Saeed S. Ahmed W. Irfan M.. (2022). Valorization of fruit peels into biovanillin and statistical optimization of process using Enterobacter hormaechei through solid-state fermentation. Fermentation, 8, 40. doi: 10.3390/fermentation8020040

[186] Medeiros A. B. P. Pandey A. Christen P. Fontoura P. S. G. de Freitas R. J. S. Soccol C. R. (2001). Aroma compounds produced by *Kluyveromyces marxianus* in solid state fermentation on a packed bed column bioreactor. World Journal of Microbiology and Biotechnology, 17, 767–771.

[187] Semenova M. G. Antipova A. S. Belyakova L. E. Polikarpov Y. N. Wasserman L. A. Misharina T. A.. (2002). Binding of aroma compounds with legumin. II. Effect of hexyl acetate on thermodynamic properties of 11S globulin in aqueous medium. Food Hydrocolloids, 16 (6), 573–584. doi: 10.1016/S0268-005X(02)00019-X)

[188] Yoon S.-H. Li C. Kim J.-E. Lee S.-H. Yoon J.-Y. Choi M.-S.. (2005). Production of vanillin by metabolically engineered *Escherichia coli*. Biotechnology Letters, 27, 1829–1832. doi: 10.1007/s10529-005-3561-4 16314978

[189] Yao W. Liu K. Liu H. Jiang Y. Wang R. Wang W.. (2021). Factories: Microbial Lipase. Front. Microbiol. 12. 10.3389/fmicb.2021.743377 PMC8489457 34616387

[190] Ayala A. Muñoz M. F. Argüelles S. (2014). Lipid peroxidation: Production, metabolism, and signaling mechanisms of malondialdehyde and 4-hydroxy-2-nonenal. Oxid. Med. Cell. Longev. 2014:360438. doi: 10.1155/2014/360438 24999379 PMC4066722

